# Significance of the CTP-binding motif for the interactions of *S. coelicolor* ParB with DNA, chromosome segregation, and sporogenic hyphal growth

**DOI:** 10.1093/nar/gkaf623

**Published:** 2025-06-30

**Authors:** Justyna Szymczak, Agnieszka Strzałka, Dominik Bania, Dagmara Jakimowicz, Marcin Jan Szafran

**Affiliations:** Department of Molecular Microbiology, Faculty of Biotechnology, University of Wrocław, 50-231 Wrocław, Poland; Department of Molecular Microbiology, Faculty of Biotechnology, University of Wrocław, 50-231 Wrocław, Poland; Department of Molecular Microbiology, Faculty of Biotechnology, University of Wrocław, 50-231 Wrocław, Poland; Department of Molecular Microbiology, Faculty of Biotechnology, University of Wrocław, 50-231 Wrocław, Poland; Department of Molecular Microbiology, Faculty of Biotechnology, University of Wrocław, 50-231 Wrocław, Poland

## Abstract

The segregation of bacterial chromosomes is widely mediated by partitioning proteins (ParAB). While ParB binds DNA specifically by recognizing short, palindromic sequences known as *parS* sites, ParA utilizes its ATPase activity to generate the force to translocate ParB–DNA nucleoprotein complexes (segrosomes). The assembly of the segrosome requires the association of ParB with *parS*, followed by nonspecific spread of the protein along the DNA. To spread on DNA, the ParB dimer must entrap the *parS* site within the complex, a process triggered by CTP binding to the conserved GERR amino acid motif. In *Streptomyces*, a genus of soil-dwelling, multigenomic bacteria that have a complex life cycle, ParB-dependent chromosome partitioning is initiated during the growth of sporogenic hyphae. However, the molecular mechanisms underlying segrosome formation in *Streptomyces* and their ability to coordinate with sporogenic development remain incompletely understood. In this study, we advance the understanding of chromosome segregation in bacteria by exploring the effects of CTP binding and hydrolysis on the formation of the partitioning complex in *Streptomyces coelicolor*. Here, via *in vitro* approaches, we demonstrate that a conserved GERR motif is essential for CTP binding and hydrolysis by *S. coelicolor* ParB. Moreover, the motif is crucial for CTP-dependent ParB accumulation on DNA. Using mutant strains, we show the significance of the GERR motif for segrosome complex assembly. Additionally, we provide data showing that the CTP-binding motif contributes to the regulation of the growth of sporogenic cells. Overall, we show that CTP-dependent segrosome assembly impacts the development of *S. coelicolor* sporogenic cells.

## Graphical Abstract

**Graphical Abstract:**
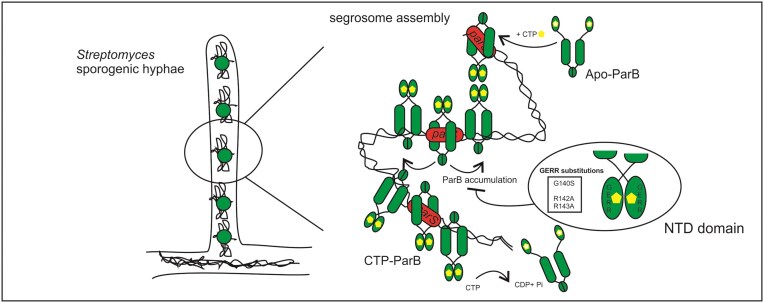

## Introduction

The faithful segregation of genetic material is a fundamental process of the cell cycle and is a prerequisite for cell propagation [1]. In most bacterial species, the segregation of chromosomes and low-copy plasmids is facilitated by a three-component system (ParABS) composed of ParA (partitioning protein A) and ParB (partitioning protein B) homologues, as well as palindromic 16-bp *parS* (*partition site*) sequences [2–4]. Depending on the species, one or several *parS* sequences are located near the *oriC* site (*origin ofchromosome replication*) [5]. The *parS* sites are recognized and bound by the ParB protein to form a large, higher-order nucleoprotein complex known as the segrosome [6–9]. Essential for specific ParB–*parS* interactions is a centrally positioned DNA-binding domain (DBD), which contains a conserved helix–turn–helix (HTH) motif. The DBD is flanked by the N-terminal (NTD) and C-terminal (CTD) domains, which are required for ParB dimerization and nonspecific DNA binding, as well as through a conserved GLGxG motif, for ParA interactions, respectively [10–17]. Additionally, the NTD has recently been demonstrated to be essential for the binding and hydrolysis of cytidine-5′-triphosphate (CTP) via the GERRxR motif, also referred to as the arginine patch (Arg patch) [18–21].

In the proposed model of bacterial chromosome segregation, the ParB dimer binds the *parS* site (in a process named ParB nucleation) and acts as a CTP molecular switch [20, 22, 23]. Upon CTP binding, the conformational changes of the ParB dimer facilitate secondary dimerization through the NTDs and promote the entrapment of DNA within a clamp-like structure of ParB named the DNA-storing chamber [22, 24]. These structural changes subsequently enable the release of the CTP–ParB dimer from the *parS* site and promote the spread across several kilobases along the DNA strand [18, 24–26]. The DNA-bound ParB can recruit other ParB dimers in a manner independent of the *parS* sequence. This feature allows ParB to cover substantial genomic distance and bypass DNA-bound roadblocks. These specific and nonspecific ParB–DNA interactions lead to DNA bridging and looping, which stimulate DNA compaction [17, 26–28]. CTP hydrolysis reopens the ParB dimer and leads to cytidine-5′-diphosphate removal and dissociation of Apo-ParB from DNA. Interestingly, several *in vitro* and *in vivo* studies have recently suggested that ParB interactions also promote phase separation of ParB–DNA condensates [28–30].

Owing to coordinated ParA and ParB activities in most bacteria, one or both segrosomes, formed on the newly replicated *oriC*, are segregated towards the opposite cell poles shortly after *oriC* duplication [2, 18]. ParA is an adenosine-5′-triphosphate hydrolase (ATPase) that binds DNA nonspecifically as an ATP–ParA dimer. The ParB–DNA complex stimulates the ATPase activity of ParA, thereby converting ParA to its monomeric, ADP-bound state, which no longer binds DNA [15, 31, 32]. This creates an ATP–ParA gradient along the chromosome, driving the transport of the CTP–ParB–DNA complex along the nucleoid through a ‘diffusion-ratchet’ mechanism [33, 34].

In *Streptomyces*, chromosome replication and active segregation are temporarily separated due to a unique lifestyle that includes sporulation [35, 36]. The *Streptomyces* life cycle starts with spore germination, followed by the apical growth and branching of elongated cells. In contrast to the process in well-studied model species, in *Streptomyces*, chromosomes are extensively replicated during vegetative growth, but cell division rarely occurs. Consequently, the vegetative hyphae are composed of adjacent, elongated cells containing multiple copies of the chromosome, similar to the mycelia of filamentous fungi. Under stress conditions or nutrient limitations, sporogenic cells develop. In these sporogenic cells, chromosomes become actively segregated, aligned along the sporogenic cell compartment, and separated with septa. These events transform multigenomic hyphal cells into chains of spores, each containing a highly compacted copy of the linear chromosome [37].

*Streptomyces* ParB binds numerous *parS* sequences (24 in *Streptomyces coelicolor* [38]) scattered near *oriC*. In the vegetative hyphae of *S. coelicolor*, ParB was shown to form nucleoprotein complexes randomly distributed in cells, except for the apical ParB complex [39–42]. Moreover, ParA localizes mostly at the apically growing hyphal tip [41]. In sporogenic cells, ParA is dispersed throughout the cell and promotes the distribution of segrosomes along the sporogenic hyphae [36]. In S*treptomyces*, the ParABS system ensures the precise positioning of multiple chromosomes along sporogenic hyphal cells and synchronizes chromosome segregation with cell elongation and multiple divisions [35]. Deletion of the *parA* and *parB* genes leads to an increased percentage of anucleate spores and the appearance of minicompartments [35, 41]. Moreover, ParA contributes to the regulation of cell extension in *Streptomyces venezuelae* [35, 43]. Despite the impact of the ParA and ParB proteins on sporogenic hyphal growth and chromosome partitioning, detailed studies on the significance of CTP for ParB-dependent chromosome segregation in *Streptomyces* have never been conducted.

Here, using a unique, multigenomic model of *S. coelicolor*, we tested how CTP binding affects segrosome assembly. Our findings demonstrated that *S. coelicolor* ParB binds and hydrolyses CTP, which is in line with recent reports on conventional model organisms. We also showed that the GERR motif is crucial for the nonspecific accumulation of the *Sc*ParB protein on DNA. Moreover, we found that the lack of CTP binding leads to the disruption of segrosome complexes in sporogenic *S. coelicolor* cells, affects their growth, and results in an increased number of segregation defects.

## Materials and methods

### Bacterial strains and plasmids

The *Escherichia coli* and *S. coelicolor* strains employed in this study are detailed in Supplementary Tables S1 and S2. The oligonucleotides and plasmids utilized are specified in  and S4. The culture conditions, antibiotic concentrations, and methods for transformation and conjugation were conducted following standard procedures for *E. coli* [44] and *S. coelicolor* [45]. All DNA manipulations were executed in accordance with standard protocols or the manufacturer’s instructions. DNA-modifying enzymes, restriction enzymes, and DNA polymerases were purchased from New England Biolabs (USA) or Thermo Fisher Scientific (USA). The oligonucleotides were synthesized by Genomed S.A. (Poland), Sigma–Aldrich (USA), or Invitrogen (USA).

### Construction of pGEX-6P-1 plasmid derivatives

The pGEX-6P-1 plasmid derivatives encoding *S. coelicolor* ParB (*Sc*ParB) variants, except the pGEX-6P-1_*parB*(HTH) derivative, were constructed on the basis of the pGEX-6P-1_*parB* vector [38]. To achieve this, custom synthesis of 325-bp DNA fragments containing specific nucleotide substitutions within the *parB* gene sequence (listed in Supplementary Table S4) was performed (Invitrogen, USA). These fragments were digested with the restriction enzymes PmlI and HindIII. Concurrently, a derivative of pGEX-6P-1_*parB* featuring an 85-bp deletion within the *parB* gene was generated by digestion with SacI, followed by plasmid religation. The religated plasmid was then digested with PmlI and HindIII, gel purified, and used for ligation to replace the shortened *parB* gene with the corresponding full-length fragment carrying a point mutation (Supplementary Table S4). To construct the pGEX-6P-1_*parB*(HTH) plasmid, a 935-bp fragment of the *S. coelicolor parB* gene was amplified by polymerase chain reaction (PCR) using the *Sc*ParB_inside_Fw and ParB_EcoRI_Rv oligonucleotides, with the H24_*parB*_HTH-*SnaBI*-*egfp* cosmid DNA [39] serving as a template. The amplified DNA fragment was digested with the restriction enzymes PmlI and NruI and subsequently ligated into the PmlI- and NruI-digested pGEX-6P-1_*parB* plasmid, yielding the pGEX-6P-1_*parB*(HTH) construct. For verification, all the pGEX-6P-1_*parB* derivatives listed in Supplementary Table S4 were tested for the presence of the designated *parB* point mutations by Sanger sequencing.

### Protein overproduction and purification

The pGEX-6P-1_*paB* derivatives were transformed into chemically competent *E. coli* BL21 (DE) pLysS cells. Positive transformants were selected on LB agar plates supplemented with ampicillin and chloramphenicol and subsequently used to overproduce *Sc*ParB variants with N-terminally fused glutathione-S-transferase (GST-*Sc*ParBs). For overproduction, selected transformants were cultured in 800 ml of LB medium supplemented with ampicillin and chloramphenicol. Overproduction of the GST-*Sc*ParB variants was induced by adding 0.5 mM isopropyl β-d-1-thiogalactopyranoside (IPTG) to exponentially growing cells, followed by incubation for 4 h at 27°C with shaking at 180 rpm. The cell culture was then centrifuged (20 min, 4°C, 4000 rpm) and the supernatant was discarded. The cell pellet was subsequently resuspended in 45 ml of buffer A (100 mM Tris–HCl, pH 8.0, 100 mM NaCl). The cells were sonicated on ice and the resulting cell lysate was centrifuged (20 min, 4°C, 10 000 rpm). The clarified lysate was incubated overnight at 4°C with 1.5 ml of buffer A-equilibrated Glutathione Sepharose^®^ 4B (GE Healthcare, USA).

Next, the GST-*Sc*ParB-bound resin was transferred to a plastic column, washed sequentially with 100 ml of buffer A and 50 ml of buffer A supplemented with 450 mM NaCl, and equilibrated with 15 ml of PreScission buffer (100 mM Tris–HCl, pH 8.0, 100 mM NaCl, 10% glycerol, 1 mM 1,4-dithiothreitol, and 1 mM ethylenediaminetetraacetic acid). The resin was then incubated overnight at 4°C with 24 units of PreScission protease (GE Healthcare, USA) to cleave the *Sc*ParB variant from the GST tag. The cleaved *Sc*ParB variants were eluted and the fractions with the highest protein concentrations were pooled. The purity of the *Sc*ParB variants was assessed via sodium dodecyl sulfate–polyacrylamide gel electrophoresis (SDS–PAGE), followed by staining with InstantBlue^®^ Coomassie protein stain (Abcam, UK). The protein concentration was determined via ROTI^®^-Quant (Carl Roth, USA). The purified *Sc*ParB variants were subsequently frozen and stored at −80°C.

### Analysis of protein–protein interactions using a bacterial two-hybrid system and β-galactosidase activity assay

To analyse *Sc*ParB dimerization, *S. coelicolor parB* gene variants were amplified via PCR using the ParB_XbaI_Fw and ParB_KpnI_Rv oligonucleotides, with pGEX-6P-1_*parB* derivatives carrying specific *parB* mutations used as templates (Supplementary Table S4). The PCR products were then purified from an agarose gel, digested with the restriction enzymes XbaI and KpnI, and ligated into XbaI- and KpnI-digested pUT18C and pKT25 vectors. The ligation products were transformed into chemically competent *E. coli* DH5α cells, resulting in a collection of pUT18C or pKT25 plasmid derivatives encoding ParB variants (as listed in Supplementary Table S4). All the constructs were verified via Sanger sequencing.

For protein–protein interaction analysis, the verified pUT18C and pKT25 derivatives encoding specific ParB variants were cotransformed into chemically competent *E. coli* BTH101 cells. The transformants were cultured on LB agar plates supplemented with ampicillin, kanamycin, 0.004% 5-bromo-4-chloro-3-indolyl-β-d-galactopyranoside, and 0.5 mM IPTG and incubated for 2 days at 30°C. Single colonies were subsequently transferred to Eppendorf tubes, resuspended in fresh LB medium, and spotted onto LB agar plates with the same supplements as those described above. After 2 days, the blue pigmentation of the colonies was assessed to verify protein interactions. For the analysis of the interactions of *Sc*ParA and *Sc*ParB, pUT18C derivatives encoding *Sc*ParB variants were cotransformed with the pKT25_*parA* vector into chemically competent *E. coli* BTH101 cells, following the procedure described above.

For the β-galactosidase activity assay, selected *E. coli* BTH101 transformants were cultured overnight at 30°C with shaking at 180 rpm in 3 ml of LB medium supplemented with ampicillin and kanamycin. The next day, 60 μl of the overnight cell cultures was used to inoculate 3 ml of fresh LB medium containing ampicillin, kanamycin, and 0.5 mM IPTG and incubated at 30°C with shaking at 180 rpm until the optical density (OD) reached ∼0.4. The cultures were then diluted five-fold with fresh LB medium and the OD was measured again. Subsequently, 2.5 ml of the diluted culture was transferred to a fresh 15-ml Falcon tube, and the β-galactosidase activity assay was performed according to a protocol described previously [46]. The β-galactosidase activity was quantified in triplicate for each *Sc*ParB variant and expressed in Miller units (Miller U), normalized to the OD of the diluted cell culture. The statistical significance of differences between *Sc*ParB variants was determined using the two-sided Student’s *t*-test.

### Analysis of protein–protein interactions using glutaraldehyde cross-linking

A glutaraldehyde cross-linking assay was performed in 15 μl samples containing 100 mM Tris–HCl (pH 8.0), 100 mM NaCl, 1 mM MgCl_2_, 0.005% Tween 20, and 4 μM of an *Sc*ParB variant. Optionally, the samples were supplemented with 4 mM CTP or 4 μM of 34-bp double-stranded DNA (dsDNA) fragment containing *parS* sequence. For cross-linking, 5 μl of glutaraldehyde solution was added to a final concentration of 0.03%, 0.06%, or 0.12%. For the control reaction, 5 μl water was added instead of glutaraldehyde. The samples were incubated at 30°C for 30 min and blocked by adding 2 μl of 2 M glycine. Next, samples were resolved via standard SDS–PAGE, followed by CBB staining with InstantBlue^®^ Coomassie protein stain. The percent contribution of dimer and high molecular weight (HMW) complex was quantified using ImageJ software by measuring the peak area and dividing it by the total signal (U) in each lane.

### Construction of *S. coelicolor* strains

To construct *S. coelicolor* strains producing *Sc*ParB variants with C-terminally fused EGFP (*Sc*ParB–EGFP), we employed the Redirect protocol, which is based on DNA homologous recombination [47]. Initially, the *vph-oriT* cassette (viomycin resistance) in the H24_*parB* HTH*-egfp* cosmid [39] was replaced with a *hyg-oriT* cassette (hygromycin resistance) via the Redirect protocol. Next, the cosmid DNA was linearized with the restriction enzyme SnaBI, purified from an agarose gel, and subsequently recombined with the PCR-amplified fragments of the *parB* gene carrying the point mutations. The *parB* gene fragments were amplified using the ParB_XbaI_Fw and ParB_KpnI_Rv oligonucleotides with the pGEX-6P-1_*parB* derivatives carrying specific nucleotide substitutions within the *parB* gene as the templates (as listed in Supplementary Table S4). The recombination of the linear PCR product and digested cosmid DNA was facilitated by cotransformation into l-arabinose-treated *E. coli* BW25113/pIJ790 cells following the Redirect protocol [47]. Positive recombinants were selected on LB agar plates supplemented with hygromycin and verified via colony PCR using the ParB_inside_Fw and ParB_inside_Rv oligonucleotides. Cosmids carrying mutations in the *parB-egfp* gene were then isolated and verified by Sanger sequencing.

The verified cosmids were subsequently used to transform chemically competent *E. coli* ET12567/pUZ8002 cells. The selected transformants were conjugated into the *S. coelicolor* J3303 strain (Δ*parB*) to replace the apramycin resistance gene (*accIV*) in the native *parB* locus with the *parB*-*egfp* variants. The exconjugants were selected on soya flour medium (SFM) supplemented with kanamycin. To identify double-crossover events, the exconjugants were restreaked on antibiotic-free SFM and screened for sensitivity to apramycin, kanamycin, and hygromycin.

### Electrophoretic mobility shift assay

To analyse the *Sc*ParB–DNA interactions *in vitro*, linear 500-bp DNA fragments containing two *parS* sites were amplified by PCR using the EMSA_*parS*_Fw and EMSA_2x_*parS*_Rv oligonucleotides. For DNA detection, the EMSA_parS_Fw oligonucleotide was custom synthesized with a 5′-conjugated cyanine (Cy) fluorophore, using cyanine 5 (Cy5) for the wild-type *parS*-containing DNA and cyanine 3 (Cy3) for the scrambled *parS-*containing DNA. The DNA templates were pUC19 plasmid derivatives containing either wild-type (pUC19_A7) or scrambled (pUC19_B2) *parS* sequences [48]. Following PCR amplification, the 5′-labelled DNA fragments were gel purified and 10 μM solutions of the fragments were prepared. These DNA fragments were then mixed with varying concentrations of *Sc*ParB variants (ranging from 200 to 1000 nM) in PreScission buffer to a final volume of 20 μl and incubated for 30 min at room temperature. After incubation, 4 μl of 70% glycerol was added to each mixture. The resulting protein–DNA complexes were resolved via electrophoresis on a 0.8% agarose gel in 1× TBE buffer at a constant voltage of 80 V for 3 h at 4°C. Free DNA and DNA–protein complexes were visualized using an Azure 600 imaging system with filters specific for detecting Cy3 or Cy5 fluorescence. The intensity of Cy5 fluorescence from free DNA was quantified using Fiji software for samples with 500 nM protein. Each experiment was performed in triplicate, and the statistical significance of differences in band fluorescence intensity was evaluated using the two-sided Student’s *t*-test.

### Biolayer interferometry

To analyse the *Sc*ParB–DNA interactions, 300-bp DNA fragments containing either a single wild-type or scrambled *parS* site were amplified by PCR using the BLI_*parS*300_Fw and BLI_*parS*300_Rv oligonucleotides. The DNA templates used for the PCR were the pUC19_A7 plasmid (containing the wild-type *parS* sequence) and the pUC19_B2 plasmid (containing the scrambled *parS* sequence). To immobilize the DNA fragments, a 5′-biotin label was added to either only the forward (one-site biotinylated DNA) oligonucleotide or both the forward and reverse (two-site biotinylated DNA) custom-synthesized oligonucleotides.

Biolayer interferometry (BLI) experiments were conducted using an Octet K2 system equipped with Octet High Precision Streptavidin (SAX) biosensors (Sartorius, USA). All the experiments were performed at 30°C. First, the streptavidin-coated biosensors were hydrated in BLI_A buffer (100 mM Tris–HCl, pH 8.0, 100 mM NaCl, 1 mM MgCl_2_, 0.005% Tween 20) for at least 10 min at room temperature. Following hydration, 150 ng of one- or two-site biotinylated dsDNA was immobilized on the biosensors in BLI_A buffer. The DNA-coated biosensors were then washed sequentially with BLI_A buffer for 60 s and BLI_B buffer (100 mM Tris–HCl, pH 8.0, 1 M NaCl, 1 mM MgCl_2_, 0.005% Tween 20, 0.1% SDS) for 300 s, and neutralized with BLI_A buffer for 300 s. Prior to each analysis, the DNA-coated biosensors were calibrated with BLI_A buffer for 60 s. For the measurements, *Sc*ParB variants were diluted in BLI_A buffer to final concentrations ranging from 100 to 1000 nM. The association and dissociation of the *Sc*ParB–DNA complex were monitored for 180 s at each step. Following each association/dissociation cycle, the biosensors were regenerated in BLI_B buffer for 300 s, neutralized with BLI_A buffer for 300 s, and then reused.

For studies examining nucleotide-dependent *Sc*ParB accumulation on DNA, *Sc*ParB (or its variant) was diluted to a concentration of 750 nM in BLI_A buffer supplemented with 1 mM nucleotide-5′-triphosphates (NTPs: CTP, dCTP, TTP, GTP, or ATP). The association and dissociation steps were then recorded as described above.

### TNP-CTP binding assay

The TNP-CTP-binding assay was performed in a final volume of 200 μl of BLI_A buffer containing 1 μM *Sc*ParB variant and 5 μM 2′,3′-*O*-trinitrophenyl-cytidine-5′-triphosphate (TNP-CTP; Jena Bioscience, Germany). Prior to measurement, the mixture was incubated for 30 min at room temperature. Optionally, the mixture was supplemented with 34-bp dsDNA containing a *parS* sequence to a final concentration of 2 μM. This DNA fragment contained either the wild-type or scrambled *parS* sequence, obtained by hybridizing two complementary 34-bp oligonucleotides: *parS*_34pz_Fw and *parS*_34pz_Rv for wild-type *parS*, or mutparS_34pz_Fw and mutparS_34pz_Rv for scrambled *parS*.

Fluorescence spectra were recorded using a Tecan Infinite 200 Pro microplate reader (Tecan Group Ltd, Switzerland) with excitation at 410 nm and emission measured in the range of 400–600 nm. The assay was conducted in a 96-well black plastic plate (Black Greiner Bio-One, Thermo Fisher Scientific, USA). For the control experiments, *Sc*ParB was subjected to heat inactivation (95°C for 10 min) prior to the addition of TNP-CTP, or wild-type *Sc*ParB was incubated with DNA containing the scrambled *parS* sequence. As a reference, the fluorescence intensity of 5 μM TNP-CTP (or 5 μM TNP-CTP supplemented with *parS*-containing DNA) was measured at a wavelength of 540 nm and normalized to 1.0 when plotted against the fluorescence intensity obtained for *Sc*ParB-containing samples (relative fluorescence) at the same wavelength. Each experiment was conducted in triplicate, and the statistical significance of the difference between the *Sc*ParB variants was determined using the two-sided Student’s *t*-test.

### CTPase activity assay

The CTPase activity of the ParB variants was quantified using an ATPase/GTPase Activity Assay Kit (Sigma–Aldrich, USA). The reaction containing 1 μM ParB variant and 4 mM CTP was performed in BLI_A buffer (100 mM Tris–HCl, pH 8.0, 100 mM NaCl, 1 mM MgCl_2_, 0.005% Tween 20) in a final volume of 40 μl. Optionally, the reaction was supplemented with 34-bp dsDNA containing the *parS* sequence to a final concentration of 2 μM. The reaction mixture was incubated for 30 min at 37°C. The absorbance at 620 nm was measured in a plastic plate (96-well SpectraPlate, Perkin Elmer, USA) using a Tecan Infinite 200 Pro, and used, according to the manufacturer’s description, to calculate the CTP hydrolysis rate as the amount of inorganic phosphate (Pi) released by the *Sc*ParB monomer per hour [Pi/h]. In the control experiment, *Sc*ParB variants were heat inactivated by incubating at a temperature of 95°C for 10 min. The measurements for each *Sc*ParB variant were performed in triplicate, and the statistical significance of the difference between *Sc*ParB variants was determined using the two-sided Student’s *t*-test.

### Circular dichroism spectroscopy

Circular dichroism (CD) spectra of the *Sc*ParB variants were measured using a J714 CD spectrometer (Jasco, Japan) equipped with a PFD-350S Peltier-type thermostat. Far-UV spectra were recorded at a temperature of 20°C in a 190–250 nm wavelength range using 50 μl of the *Sc*ParB variant in PreScission buffer and a quartz cuvette with a path length of 10 mm. Each CD spectrum recording was performed in triplicate, and the average ellipticity value was normalized against the molar protein concentration [molar ellipticity (deg cm^2^ /dmol)] and plotted versus the wavelength (nm).

### Epifluorescence and structured illumination microscopy

For the microscopic analysis of chromosome segregation, *S. coelicolor* cultures were prepared on glass coverslips inserted into SFM agar plates. The hyphae that grew attached to the coverslip were stained with DAPI and wheat germ agglutinin (WGA) conjugated to Texas Red (WGA-TexasRed) following a protocol described previously [41], with the exception that cell fixation was carried out by washing the glass-attached mycelia three times with absolute methyl alcohol (or optionally with a 2.8% paraformaldehyde solution as described earlier by Jakimowicz *et al.* [38]). Standard epifluorescence observations of the nucleoid (DAPI-stained), cell wall (WGA-TexasRed-stained), or *Sc*ParB–EGFP variants were conducted using a Leica LAS X Widefield microscope equipped with a Fluotar 100×/1.32 objective. The exposure times for the respective fluorophores were as follows: DAPI, 40 ms; Texas Red, 200 ms; and EGFP, 200 ms. The length of prespores was measured as the distance between neighbouring septa. The prespore compartments <0.5 μm were categorized as minicompartments. Chromosome segregation defects were measured as the percentage of prespore compartments lacking DAPI fluorescence. A total of 510 prespore compartments were analysed for each *S. coelicolor* strain. Additionally, to assess the length of sporogenic hyphae, the distance from the hyphal tip to the basal septum was measured in 50 sporogenic hyphae with clearly visible septa.

For structured illumination microscopy (SIM) observations, *S. coelicolor* cultures were prepared on coverslips and methanol-fixed as described above, with the exception that, to visualize the cell wall, hyphae were stained with WGA conjugated to Alexa Fluor 633 (WGA-AF633) diluted to a final concentration of 2.5 μg/ml in phosphate buffered saline. SIM observations were conducted using a Zeiss Elyra 7 microscope 467 equipped with an Alpha Plan-APO 100×/1.46 Oil DIC VIS lens, sCMOS + emCCD camera and Andor EM-CCD 468 camera. Excitation of the EGFP and AF633 fluorophores was performed at wavelengths of 488 and 632 nm, with 10% and 5% of the laser power applied, 5000 and 500 mW, at 488 and 632 nm, respectively. The exposure time was set to 50 ms for both fluorophores. A series of 15–37 cross-sections (Z-stacks) were collected, with a 100 nm distance between frames. The data were preprocessed using Lattice SIM image reconstruction algorithm implemented in Zeiss Zen software, followed by bioinformatics analyses with ImageJ software equipped with Fiji 3D Image Suite [49].

*Sc*ParB–EGFP complexes were analysed in 18–19 hyphal images with clearly detectable sporogenic septa, which served as markers for *S. coelicolor* sporogenic development. To quantify the *Sc*ParB–EGFP foci, selected regions of the sporogenic hyphae were segmented using the Fiji 3D Image Suite package to generate a 3D view of the *Sc*ParB–EGFP complexes. Initially, local fluorescence signal maxima were identified using the 3D ‘Local Maxima’ function. These maxima were then used as seeds for segmentation with 3D spot segmentation with a Gaussian model function. The segmentation parameters were set as follows: threshold = 10 000; radius = 50; and standard deviation (SD) = 1.75. The parameters were selected to best reflect the number of EGFP complexes identified by eye inspection in the *S. coelicolor* strain producing wild-type *Sc*ParB–EGFP, and were then applied to the analysis of other strains producing particular *Sc*ParB variants. The threshold parameter determined the number of seeds used by the segmentation function. Radius and SD parameters were set up to calculate the radial Gaussian model around each seed (radius) and to compute the individual threshold for each complex (SD). SD = 1.75 ensured that up to 92% of the Gaussian curve was included in the further analysis. *R* was used to visualize the volume and fluorescence intensity of the detected complexes.

## Results

### CTP binding and hydrolysis are stimulated by the ScParB–*parS* interactions

**Figure 1.**
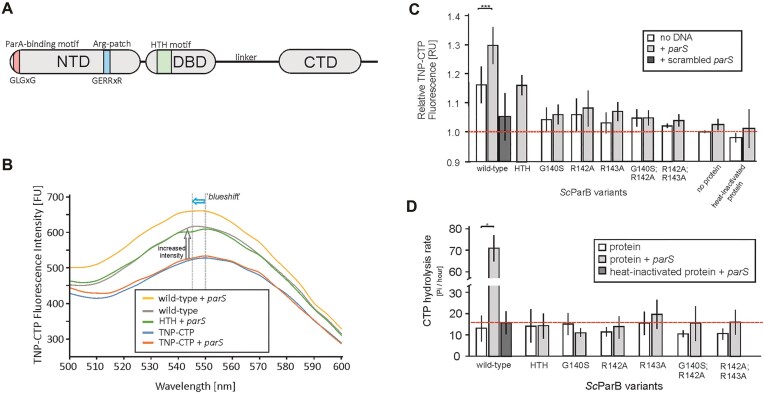
CTP binding and hydrolysis by *Sc*ParB. (**A**) Schematic representation of the subdomain organization of the *S. coelicolor* ParB protein, highlighting the NTD containing the ParA-binding site (GLGxG), the conserved arginine patch (Arg patch, GERRxR), the DBD featuring an HTH structural motif, and the C-terminal dimerization domain (CTD), which is connected to the DBD via a flexible linker. (**B**) Representative fluorescence (FU) spectrum of 5 μM TNP-CTP after binding to either wild-type *Sc*ParB or *Sc*ParB^HTH^ (1 μM) in the presence or absence of a 34-bp DNA fragment (2 μM) containing the *parS* sequence. In the control experiments, the fluorescence of 5 μM TNP-CTP and TNP-CTP supplemented with 2 μM 34-bp *parS*-containing DNA was measured. The grey dotted lines indicate the maxima of the fluorescence spectra. The arrows denote the increase in light emission (grey) or the ‘blueshift’ in the emission spectrum (blue) following TNP-CTP binding. (**C**) The average relative fluorescence response units (RUs) of 5 μM TNP-CTP when incubated with 1 μM *Sc*ParB variants (wild type, G140S, R142A, R143A, G140S;R142A, R142A;R143A, and HTH) in the absence (white) or presence of the 2 μM 34-bp DNA fragment containing the *parS* (grey) or scrambled *parS* (dark grey) site. The fluorescence of unbound 5 μM TNP-CTP was measured at a wavelength of 540 nm, set to 1.0 and marked with a red dotted line. (**D**) CTP hydrolysis rate, expressed as μmole of Pi released per μmoles of *Sc*ParB molecules per hour. The analysis was conducted in the presence of 1 μM *Sc*ParB variants (wild type, G140S, R142A, R143A, G140S;R142A, R142A;R143A, and HTH) and 4 mM CTP, and in the absence (white) or presence (grey) of 2 μM 34-bp *parS*-containing DNA. In a control experiment, *Sc*ParB variants were heat inactivated prior to fluorescence detection (dark grey). The hydrolysis rate of wild-type *Sc*ParB in the absence of *parS*-containing DNA is marked with a red dotted line. All experiments were performed in triplicate, and the quantified standard deviations are indicated. Statistical significance was determined via Student’s *t*-test: *P*-value <.05 (*), <.01 (**), and <.001 (***).

The NTD of *S. coelicolor* ParB (*Sc*ParB), similar to that of other ParB homologues, contains a conserved GERRxR amino acid sequence previously shown to bind CTP (Fig. 1A and Supplementary Fig. S1). To study the impact of CTP on ParB activity in *S. coelicolor*, we first overproduced and purified a set of *Sc*ParB variants carrying amino acid substitutions within the GERR motif. The modifications included single (*Sc*ParB^G140S^, *Sc*ParB^R142A^, and *Sc*ParB^R143A^) or double amino acid substitutions (*Sc*ParB^G140S;R142A^ and *Sc*ParB^R142A;R143A^). These substitutions did not affect the overall spatial structure of the *Sc*ParB variants, as confirmed by CD spectroscopy (Supplementary Fig. S2).

Next, we tested the binding of the *Sc*ParB variants for a fluorescently labelled CTP analogue, TNP-CTP. The accommodation of TNP-CTP in the binding pocket of *Sc*ParB resulted in an increase in the fluorescence signal (RU) and shifted the maximum emitted fluorescence signal towards a shorter wavelength (blueshift) (Fig. 1B and Supplementary Fig. S3). Thge relative fluorescence signal of TNP-CTP increased when the nucleotide was incubated with wild-type *Sc*ParB (1.16 ± 0.06 RU) compared with that in the control experiments with no protein added (1.00 ± 0.00 RU) or when the wild-type *Sc*ParB was first heat inactivated (0.98 ± 0.02 RU) (Fig. 1C). For all the GERR-substituted *Sc*ParB variants, the detected TNP-CTP fluorescence (from 1.02 to 1.07 RU) was comparable to that of the negative controls (Fig. 1C), indicating that an intact GERR motif is required for CTP binding.

Having established that *Sc*ParB binds CTP, the next step was to investigate whether the formation of the ScParB–*parS* complex affects the affinity of *Sc*ParB for the nucleotide. To this end, we incubated the wild-type *Sc*ParB or GERR-substituted variants with TNP-CTP and a 34-bp-long DNA fragment containing a single *parS* sequence. We observed that the addition of *parS*-containing DNA stimulated TNP-CTP binding. The relative fluorescence signal in the presence of wild-type *Sc*ParB (1.29 ± 0.06 RU) was significantly increased than the signal in the absence of DNA (1.16 ± 0.06 RU) or in the presence of a scrambled *parS* sequence (1.04 ± 0.08 RU) (Fig. 1C). As predicted, for all GERR-substituted *Sc*ParB variants, which did not interact with TNP-CTP, the relative fluorescence signals in the presence or absence of DNA were comparable (Fig. 1C). The increased affinity of *Sc*ParB for TNP-CTP in the presence of *parS* was not detected for the non-DNA-interacting *Sc*ParB^HTH^ variant. The TNP-CTP relative fluorescence signal (1.16 ± 0.03 RU) for this variant in the presence of *parS* was comparable to that of wild-type *Sc*ParB without the addition of *parS* (1.16 ± 0.06 RU) (Fig. 1C). This finding indicates that although *Sc*ParB binds CTP *in vitro* in the absence of *parS*-containing DNA, its affinity for the nucleotide is increased when *Sc*ParB binds *parS*.

Next, we tested whether the presence of the *parS* sequence impacts not only CTP binding but also CTP hydrolysis (CTPase activity). For the wild-type *Sc*ParB protein in the absence of *parS*, the CTP hydrolysis rate was low, with 13.0 ± 4 Pi/h released per ParB monomer. Since a similar CTP hydrolysis rate was detected for heat-inactivated *Sc*ParB (15.6 ± 6 Pi/h), we estimated the rate quantified above as spontaneous CTP degradation under the assay conditions. When the reaction mixture was supplemented with *parS*-containing DNA, the CTPase activity of wild-type *Sc*ParB notably increased to 71.1 ± 6 Pi/h (Fig. 1D). As predicted, a lack of CTPase activity (8.4–19.4 Pi/h) was observed for the *Sc*ParB variants with GERR motif substitutions, which is consistent with their lack of CTP binding (Fig. 1D). Moreover, the CTPase activity of *Sc*ParB^HTH^, which interacts with CTP but not with DNA (Supplementary Fig. S4A), was completely abolished (14.1 ± 6 Pi/h). These findings confirm that the interaction of *Sc*ParB with *parS* increases its CTPase activity.

Taken together, our observations confirmed that *S. coelicolor* ParB is a CTP-binding protein. CTP binding is facilitated by interactions with glycine and two arginine residues within the conserved GERR motif located in the NTD. Moreover, our findings also indicate that the CTP–*Sc*ParB complex can be formed in the absence of *parS*, but nucleotide binding is stimulated as a result of the ScParB–*parS* interaction. The interaction of the CTP–*Sc*ParB complex with *parS* is required for the CTPase activity of the protein–nucleotide complex.

### CTP does not contribute to the association of *Sc*ParB with *parS*

**Figure 2.**
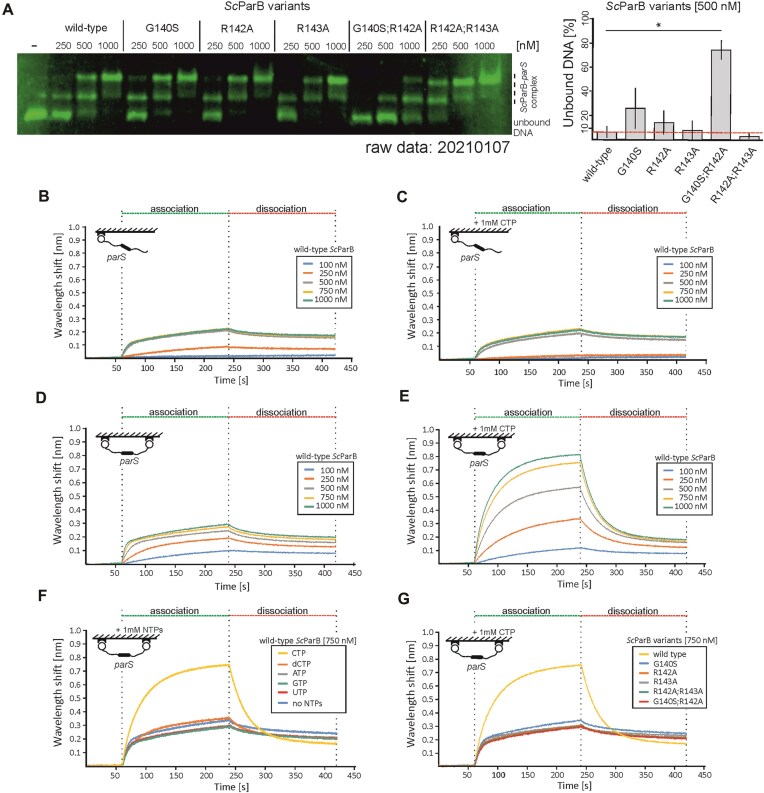
Impact of CTP on *Sc*ParB–DNA interactions *in vitro*. (**A**) Lef: Analysis of the binding of recombinant *Sc*ParB variants (wild type, G140S, R142A, R143A, G140S;R142A, and R142A;R143A) to a 500-bp Cy5-labelled linear DNA fragment (10 μM) containing two *parS* sites. The *Sc*ParB concentrations ranged from 250 to 1000 nM. Right: The unbound DNA fraction (%) was quantified with an *Sc*ParB variant concentration of 500 nM. The experiment was conducted in triplicate, with the quantified standard deviations indicated. Statistical significance was determined via Student’s *t*-test: *P* values <.05 (*), <.01 (**), and <.001 (***). (**B**) BLI-measured binding of wild-type *Sc*ParB (100–1000 nM protein) to a one-end biotin-immobilized 300-bp DNA fragment containing a wild-type *parS* site, also (**C**) conducted in the presence of 1 mM CTP. (**D**) BLI-measured binding of wild-type *Sc*ParB (100–1000 nM protein) to a two-end biotin-immobilized 300-bp DNA fragment containing a wild-type *parS* site, also (**E**) conducted in the presence of 1 mM CTP. (**F**) BLI-measured binding of 750 nM wild-type *Sc*ParB to a two-end biotin-immobilized 300-bp DNA fragment containing a wild-type *parS* site performed in the presence of various nucleotide-5′-triphosphates (CTP, dCTP, ATP, GTP, or UTP) at 1 mM. (**G**) BLI-measured binding of 750 nM *Sc*ParB variants (wild type, G140S, R142A, R143A, G140S;R142A, and R142A;R143A) to a two-end biotin-immobilized 300-bp DNA fragment containing a wild-type *parS* site performed in the presence of 1 mM CTP. The association and dissociation steps are indicated by green and red dotted lines, respectively.

Given that the GERR motif is essential for CTP binding and hydrolysis, we tested whether the absence of CTP binding affects the interactions of *Sc*ParB with DNA. First, via an electrophoretic mobility shift assay (EMSA), we examined the interactions of *Sc*ParB variants with a 500-bp-long, Cy5-labelled DNA fragment containing two *parS* sites (compared with Cy3-labelled DNA with scrambled *parS*). The wild-type *Sc*ParB bound specifically to *parS*-containing DNA at the lowest tested concentration (250 nM) (Fig. 2A), discriminating against the scrambled *parS* sites used in the control assay (Supplementary Fig. S4A). At 250 nM *Sc*ParB, only a single *Sc*ParB–*parS* complex was detected, whereas at 500 nM, two separate ParB–*parS* complexes were observed, and the amount of free DNA remained below 10%. Since no large protein complex was detected, this observation suggests that the association of *Sc*ParB with both *parS* sequences (*Sc*ParB nucleation) may occur independently (Fig. 2A). Additionally, the presence of 1 mM CTP in the reaction mixture did not affect the assembly of the protein–DNA complex, suggesting that CTP has no effect on *Sc*ParB nucleation on *parS* sequences (Supplementary Fig. S4B). All the tested *Sc*ParB variants interacted with *parS*-containing DNA with a similar affinity to that of the wild-type *Sc*ParB, except for the *Sc*ParB^G140S;R142A^ variant, the DNA binding of which was diminished, resulting in a greater fraction of unbound DNA at a 500 nM protein concentration (Fig. 2A). As expected, the presence of CTP did not affect the *parS* binding of the GERR-substituted *Sc*ParB variants (Supplementary Fig. S4C).

The results of the EMSAs were further confirmed by detailed analysis of the *Sc*ParB–DNA interactions using BLI. In this experiment, we used a 300-bp *parS*-containing DNA fragment biotinylated at the 5′-end and immobilized on the BLI sensor surface (Fig. 2B, inset). The binding of wild-type *Sc*ParB to DNA was observed at a protein concentration of 250 nM, whereas at a concentration of 500 nM (or higher), the detected signals were comparable and reached a plateau (Fig. 2B). The dissociation constant (*K*_d_) of the ScParB–*parS* complex for one-end immobilized DNA was 271 ± 14 nM (Supplementary Fig. S5). The addition of 1 mM CTP to the association buffer did not significantly affect the association curve, and the saturation of *parS* sites was achieved at protein concentrations comparable to those under CTP-free conditions (Fig. 2C). This observation reinforces the EMSA result indicating that CTP is not required for *Sc*ParB–*parS* binding.

### CTP binding is required for *Sc*ParB accumulation on DNA

Given that no high-molecular-weight protein–DNA complex was detected in our EMSA or BLI studies, we further investigated the affinity of *Sc*ParB for the DNA substrate that enables the spread of ParB. To this end, as in the above-described experiment, a 300-bp-long DNA fragment was attached to the surface at both ends, creating two spatial ‘roadblocks’ with a single *parS* site located between them (Fig. 2D, inset). BLI analyses revealed that in the absence of CTP, *Sc*ParB bound to *parS*-containing DNA attached at two ends (Fig. 2D) with an affinity comparable (167 ± 27 nM; Supplementary Fig. S5) to that observed for DNA with one end immobilized (Fig. 2B and Supplementary Fig. S5). Next, we compared the interaction of *Sc*ParB with *parS*-containing two-end immobilized DNA in the presence of 1 mM CTP (Fig. 2E). The addition of 1 mM CTP strongly stimulated *Sc*ParB binding to DNA with both ends immobilized, which suggested the accumulation of the protein on the DNA. The CTP-dependent accumulation of *Sc*ParB on DNA was observed even at the lowest tested CTP concentration (63 μM) and increased at higher CTP concentrations (Supplementary Fig. S6A). Further analysis confirmed that *Sc*ParB accumulation on DNA occurs specifically only in the presence of the *parS* site, as it was not detected for the *Sc*ParB^HTH^ variant or on DNA with a scrambled *parS* site (Supplementary Fig. S6B–D). The accumulation of *Sc*ParB on DNA was also not observed in the presence of other nucleotide-5′-triphosphates (ATP, GTP, or UTP) or deoxycytidine-5′-triphosphate (dCTP), indicating the strong specificity of the CTP-binding pocket for the coordinated nucleotide (Fig. 2F). The impact of CTP on *Sc*ParB–DNA interactions was further verified by analysing *Sc*ParB variants with GERR motif substitutions. Although these *Sc*ParB variants interacted with *parS*-containing DNA, they were unable to accumulate on DNA in the presence of CTP (Fig. 2G), confirming the significance of CTP binding for *Sc*ParB accumulation on DNA.

In summary, our observations reinforce the model proposed for ParB homologues in other bacterial species, which posits that nonspecific *Sc*ParB accumulation on DNA requires both the presence of *parS* sequences and CTP binding. We infer that the observed accumulation reflects *Sc*ParB spreading from the *parS* site.

### Lack of CTP binding by *Sc*ParB affects segrosome assembly in *S. coelicolor*

**Figure 3.**
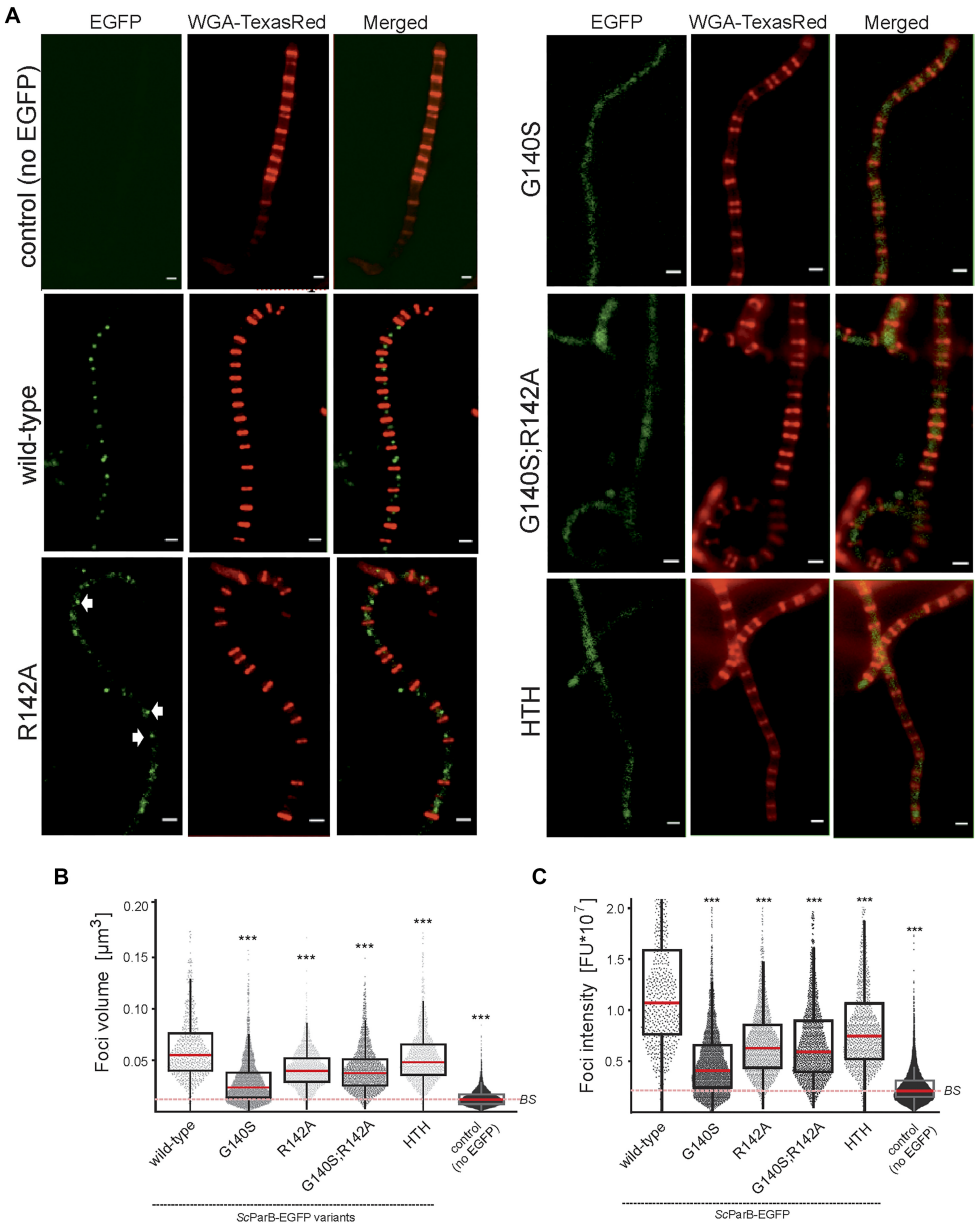
Effect of GERR motif substitution on *Sc*ParB–EGFP complex assembly. (**A**) Visualization of fluorescence foci in methanol-fixed *S. coelicolor* sporogenic hyphae of strains producing *Sc*ParB–EGFP (wild type, G140S, R142AP, G140S;R142A, and HTH) using standard epifluorescence microscopy. The peptidoglycan was visualized using WGA conjugated to Texas Red (WGA-TexasRed). The wild-type *S. coelicolor* strain, which does not produce EGFP-fused *Sc*ParB, served as a control. Scale bar: 1 μm. Box plots representing the volume (**B**) and intensity (**C**) of fluorescence foci in strains producing *Sc*ParB–EGFP variants, as identified using SIM microscopy. Each box plot shows the median (red) with the first and third quartiles, whereas the lower and upper ‘whiskers’ extend to values no further than 1.5 times the interquartile range. Statistical significance was determined via Student’s *t*-test: *P* values <.05 (*), <.01 (**), and <.001 (***). The baseline signal (BS) is indicated with a red dotted line.

Given that CTP binding promotes *Sc*ParB accumulation on DNA, we next investigated how the elimination of CTP binding influences segrosome formation in *S. coelicolor* sporogenic hyphae, i.e. at the stage of segregation of multiple chromosomes into unigenomic prespores. For this purpose, we constructed *S. coelicolor* strains producing GERR-substituted *Sc*ParB variants, each C-terminally fused with EGFP (*Sc*ParB^G140S^–EGFP, *Sc*ParB^R142A^–EGFP, and *Sc*ParB^G140S;R142A^–EGFP). These strains were compared to those producing the wild-type *Sc*ParB–EGFP or its non-DNA-interacting *Sc*ParB^HTH^–EGFP variants (Supplementary Fig. S7). Despite numerous attempts, we were not successful in the selection of *S. coelicolor* strains producing R143A-substituted *Sc*ParB variants, namely, *Sc*ParB^R143A^–EGFP and *Sc*ParB^R142A;R143A^–EGFP. First, we examined the formation of segrosomes in methanol-fixed *S. coelicolor* cultures producing *Sc*ParB–EGFP variants using standard epifluorescence microscopy (Fig. 3A and Supplementary Fig. S8). In the strain producing the wild-type *Sc*ParB–EGFP variant, bright and uniformly spaced *Sc*ParB–EGFP foci were observed between the septa, forming chains of prespores, as described previously [39, 50]. In contrast, in the strain producing *Sc*ParB variant R142A-EGFP, the fluorescence foci were rare and appeared randomly. However, in other *Sc*ParB–EGFP variants, also deficient in CTP binding, namely *Sc*ParB^G140S^–EGFP and *Sc*ParB^G140S;R142A^–EGFP, the fluorescence signal was highly diffuse along the sporogenic hyphae, similar to the fluorescence signal observed in the strain producing *Sc*ParB^HTH^–EGFP, in which DNA binding was abolished.

High-resolution SIM microscopy was subsequently employed to further characterize the *Sc*ParB–EGFP foci. SIM analyses allowed measurement of both the intensity and volume of methanol-fixed *Sc*ParB–EGFP complexes in the studied *S. coelicolor* strains (Supplementary Figs S9 and S10). The average volume of the wild-type *Sc*ParB–EGFP complexes was 0.062 μm^3^, reaching up to 0.17 μm^3^ (Fig. 3B), with a mean intensity of 1.2 × 10^7^ fluorescence units (FU), which was significantly greater than that detected in the other strains (Fig. 3C). In contrast, the *Sc*ParB foci detected in the strain producing the ParB^G140S^-EGFP variant had both the smallest volume (0.029 μm^3^) and the lowest fluorescence intensity (0.4 × 10^7^ FU). However, SIM image processing detected foci of the GERR-substituted *Sc*ParB–EGFP variants also formed numerous faint and/or small foci (Fig. 3B and C). The SIM analyses also revealed foci in the strain producing *Sc*ParB^HTH^–EGFP, with an average focus volume of 0.054 μm^3^, reaching up to 0.17 μm^3^ with a mean intensity of 0.8 × 10^7^ FU. Both the average focus volume and intensity of *Sc*ParB^HTH^–EGFP were significantly lower than those detected for the wild-type *Sc*ParB–EGFP. Intriguingly, their volume and intensity were slightly greater than those of the GERR-substituted *Sc*ParB–EGFP variants. Notably, autofluorescence was also detected in the wild-type *S. coelicolor* M145 strain, which does not produce the EGFP-fused ScParB protein with the average focus volume of 0.012 μm³ and intensity of 0.2 × 10^7^ FU (background signal, BS). However, the autofluorescence was significantly lower than that in *S. coelicolor* strains producing EGFP-fused *Sc*ParB variants (Fig. 3C). Thus, SIM analyses confirmed that the GERR substitutions reduced the ability of ParB-EGFP to form the large and bright foci.

Surprisingly, when we verified the impact of the G140S substitution on the *Sc*ParB–ScParB interaction using the bacterial two-hybrid (BACTH) system and β-galactosidase activity assay, we noticed that the *Sc*ParB^G140S^ and *Sc*ParB^G140S;R142A^ variants were also defective in ParB dimerization but not in the interaction with *Sc*ParA (Supplementary Fig. S11A). Intriguingly, the inefficient dimerization of *Sc*ParB^G140S^ variant was not confirmed by its glutaraldehyde cross-linking *in vitro*. In comparison to the wild-type *Sc*ParB, the *Sc*ParB^G140S^ variant retained, although slightly lower (3.1% versus 1.1% of dimers detected for the wild-type and G140S *Sc*ParB variants, respectively), its capacity for protein–protein interactions as dimers or HMW complexes were detectable in CBB-stained gel (Supplementary Fig. S11B). Interestingly, we also noticed that the addition of CTP or *parS* containing 34-bp dsDNA slightly stimulated dimerization of the wild-type *Sc*ParB (6.4% or 5.3% of dimer depending on the cross-linking conditions) but not G140S-subsituted variant (1.6% or 2.1% of dimer) *in vitro* (Supplementary Fig. S11B).

On the other hand, the R142A substitution had the opposite effect and resulted in up to three-fold enhancement of the dimerization of the *Sc*ParB^R142A^ variant when analysed using BACTH analysis (Supplementary Fig. S11A) or protein cross-linking assay *in vitro* (Supplementary Fig. S11C) than with that of wild-type *Sc*ParB. The increased dimerization of the R142A-substituted variant may explain its formation of fluorescent foci.

In summary, using EGFP-tagged *Sc*ParB variants, we demonstrated that the lack of CTP binding significantly affects segrosome assembly during sporulation of *S. coelicolor*. Substitutions within the GERR motif result in less efficient assembly of the *Sc*ParB–DNA complexes and/or their misplacement in sporogenic cells. Moreover, we showed that modifications within the GERR motif can also impact the ability of *Sc*ParB to dimerize, which is stimulated by the CTP or *parS* binding.

### Substitutions within the GERR motif affect *S. coelicolor* sporulation

**Figure 4.**
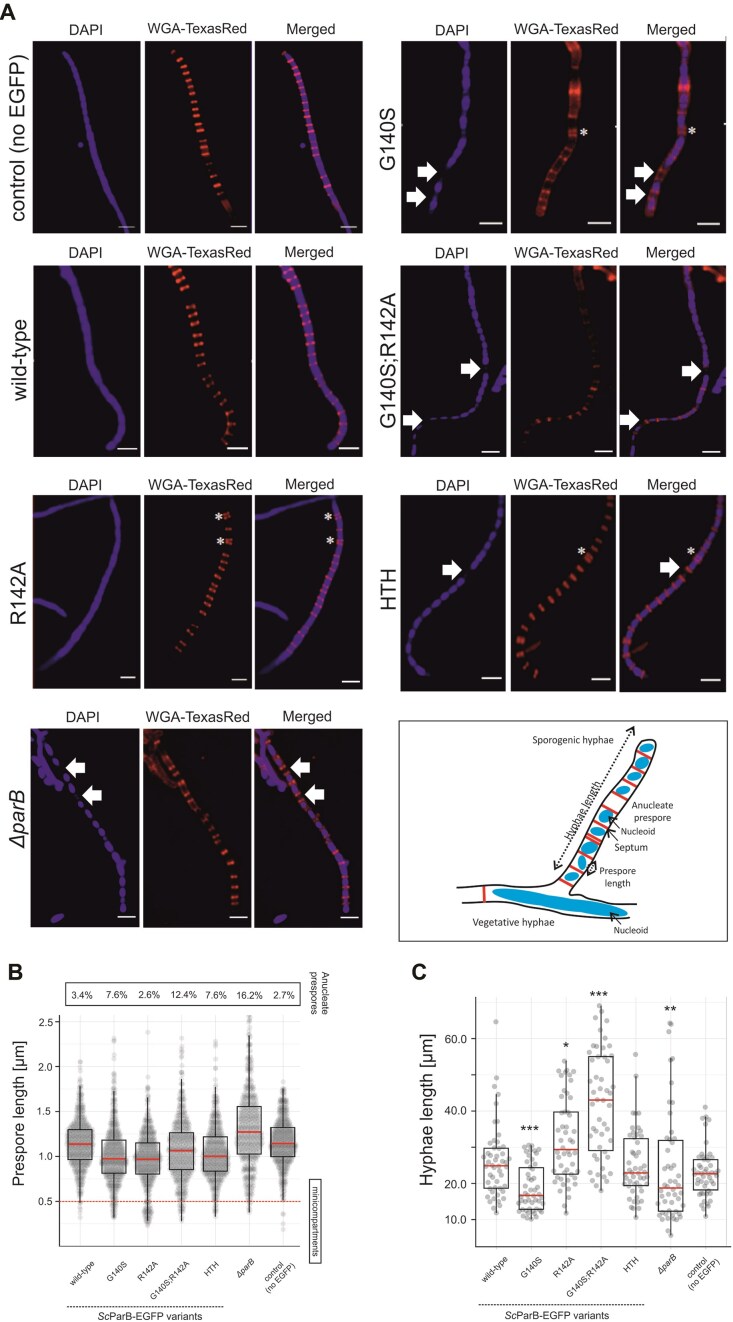
Effect of GERR substitutions in *Sc*ParB–EGFP on *S. coelicolor* chromosome segregation and prespore formation. (**A**) Localization of nucleoids (DAPI-stained) and peptidoglycan (stained with WGA, WGA-TexasRed) in methanol-fixed *S. coelicolor* strains producing *Sc*ParB–EGFP variants (wild-type, G140S, R142A, G140S;R142A, and HTH) or lacking the *parB* gene (Δ*parB*). The wild-type *S. coelicolor* strain, which does not produce the EGFP-fused *Sc*ParB protein, served as a control. Asterisks indicate the positions of the minicompartments (<0.5 μm) and arrows highlight prespores lacking DAPI fluorescence. Scale bar: 2 μm. The scheme shows the key elements of sporogenic hyphae morphology analysed in this study. (**B**) Box plots displaying the distribution of prespore lengths (*n* = 510 for each strain). The red dotted lines indicate minicompartments. The percentage of prespore compartments lacking a DAPI signal is shown above. (**C**) Box plot analysis of the lengths of sporogenic hyphae in *S. coelicolor* strains producing *Sc*ParB–EGFP variants (wild-type, G140S, R142A, G140S;R142A, and HTH) or lacking the *parB* gene (Δ*parB*). The wild-type *S. coelicolor* strain, which does not produce EGFP-fused *Sc*ParB, served as a control. The length from the hyphal tip to the farthest detectable septum was measured on the basis of 50 images of sporogenic hyphae collected from each strain. All box plots show the median (red) with the first and third quartiles, whereas the lower and upper ‘whiskers’ extend to values no further than 1.5 times the interquartile range. Statistical significance was determined via Student’s *t*-test: *P-*values <.05 (*), <.01 (**), and <.001 (***).

Previous studies [42] as well as our analysis have shown that *parB* deletion in *S. coelicolor* increases the percentage of anucleate spores up to 16.2% and results in irregularly laid septa (Fig. 4). Here, we also revealed that the formation of *Sc*ParB–DNA complexes *in vitro* and *in vivo* was strongly affected by the elimination of CTP binding caused by GERR motif substitutions. Therefore, to further assess the phenotypic effects of the introduced mutations, we analysed their impact on chromosome segregation and septation in *S. coelicolor* sporogenic hyphae. To this end, we used a set of previously characterized *S. coelicolor* strains producing wild-type *Sc*ParB and GERR-substituted *Sc*ParB variants fused with EGFP. These strains were stained with DAPI to visualize the nucleoids and with WGA-TexasRed to detect sporogenic septa (Fig. 4A). Detailed analysis of prespore length revealed that, compared with the wild-type ScParB–EGFP-producing strain (or the control strain with no EGFP fusion), only the *S. coelicolor* strains producing *Sc*ParB variants with the R142A substitution (including the double G140S;R142A substitution) presented an aberrant distance between septa and an increased number of minicompartments (prespore length <0.5 μm) (Fig. 4B). Moreover, the introduced mutations led to the formation of prespore compartments lacking nucleoids. The percentage of anucleate prespores was 2.7% and 3.4% in both control strains producing the wild-type *Sc*ParB protein and those producing the EGFP-fused variant, respectively. In the strains producing the *Sc*ParB^G140S^–EGFP or non-DNA-interacting *Sc*ParB^HTH^–EGFP variant, the number of anucleate prespores was elevated to 7.6%. On the other hand, the production of the *Sc*ParB^G140S;R142^–EGFP variant increased the number of prespores lacking DNA to 12.5% (Fig. 4B). Intriguingly, the fraction of anucleate spores in all tested *S. coelicolor* strains was lower (from 2.7% to 12.4%, depending on the strain) than detected in the *parB* deletion genetic background (Fig. 4).

Next, since ParA and ParB were shown to affect the extension of sporogenic cells [35], we analysed the length of sporogenic cells in the constructed mutant strains. The production of the G140S-substituted variant resulted in the formation of significantly shorter sporogenic hyphae (18.7 ± 6.4 μm) than those produced by the strains producing the wild-type *Sc*ParB (with or without EGFP fusion, 26.1 ± 10.1 and 22.6 ± 7.4 μm, respectively) or *Sc*ParB^HTH^ (25.2 ± 9.8 μm) (Fig. 4C). On the other hand, in the strain producing *Sc*ParB^R142A^, the length of sporogenic hyphae increased to 32.0 ± 11.4 μm, whereas this increase was even more pronounced in the strain producing *Sc*ParB^G140S;R142A^ (42.1 ± 14.6 μm). Interestingly, the elongated sporogenic hyphae detected in the strain producing *Sc*ParB^G140S;R142A^ corroborate the observation that the growth of the strain in liquid medium was also accelerated compared with that of the wild-type strain (or strains with single GERR motif substitutions) (Supplementary Fig. S12).

In summary, our results showed that abolishing of CTP binding by *Sc*ParB affects the development of sporogenic cells and disturbs chromosome segregation.

## Discussion

Here, we showed that the *S. coelicolor* partitioning protein ParB binds and hydrolyses CTP in a *parS*-dependent manner. Moreover, CTP binding, which is mediated by the GERR motif located within the NTD, is essential for *Sc*ParB accumulation on DNA and segrosome complex formation.

**Figure 5.**
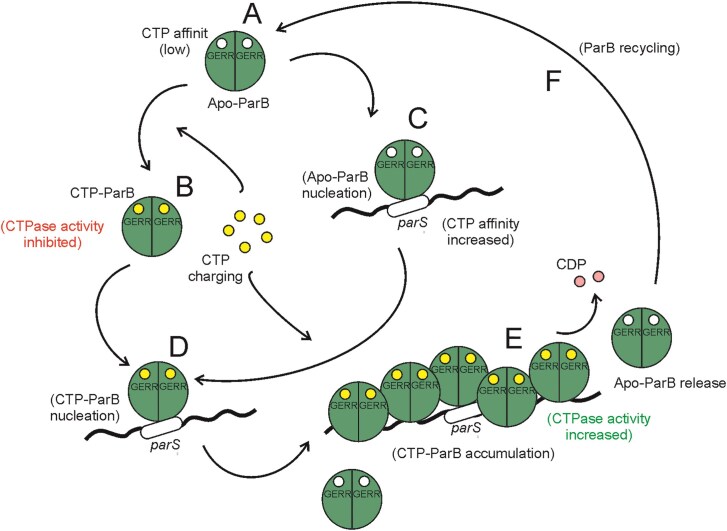
Model of *Sc*ParB interactions with DNA.

In all the proposed models, the first step of segrosome assembly is the specific binding of ParB to the *parS* site [4, 22, 23]*. Streptomyces coelicolor* ParB has been reported to form a complex with *parS* with a dissociation constant (*K*_d_) that varies from 33 [51] to 480 nM [38]. However, our findings did not corroborate these observations, as the BLI-estimated *K*_d_ values ranged from 176 to 271 nM, depending on the immobilized DNA. We demonstrated that in *S. coelicolor*, both Apo-*Sc*ParB and CTP–*Sc*ParB exhibit similar affinities for the *parS* site, suggesting that the nucleation step is CTP independent (Fig. 5A–D). To our knowledge, the intracellular CTP levels in *S. coelicolor* have not been determined. However, the CTP concentration in other bacteria, which is 10-fold greater than the *K*_d_ of the CTP–ParB complex (∼10 μM), suggests preloading of *Sc*ParB with CTP (CTP–*Sc*ParB) before it binds to *parS* [19]. Intriguingly, CTP binding to *Caulobacter crescentus* ParB (*Cc*ParB) was not detected in the absence of *parS*-containing DNA or was found to be very low for *B. subtilis* (*Bs*ParB) [18, 19].

We speculate that, at least in *Streptomyces*, the initial step of *Sc*ParB nucleation is independent of nucleotide availability, as all tested GERR-substituted variants bind *parS*-containing DNA with affinity comparable to that of the wild-type protein. Our observations are also supported by studies on the *Cc*ParB homologue, in which the *Cc*ParB^G101S^ and *Cc*ParB^R104A^ variants were able to nucleate at the *parS* site [52]. In contrast to the interaction in *C. crescentus* or *B. subtilis*, the association of *Sc*ParB with the *parS* site is not essential for CTP binding, as both the wild-type *Sc*ParB and the ScParB^HTH^ variant, which is defective in *parS* interaction, exhibit similar strengths in nucleotide binding. However, when *Sc*ParB was bound to the *parS* site, its affinity for CTP increased, suggesting that *Sc*ParB was immediately charged with CTP after the association with DNA. These findings align with those of hydrogen–deuterium exchange studies on *Mx*ParB, which demonstrated that structural changes occurring after *Mx*ParB nucleation at the *parS* site can be transmitted across the protein, affecting the GERR motif within the NTD [20]. The NTD contributes to secondary ParB dimerization, known as N-gate closure, which is stimulated by CTP, particularly in the presence of the *parS* motif. The CTP-dependent intermolecular association of NTDs is crucial for the transformation of the ParB dimer into a clamp-like structure that encircles the *parS* site [24], facilitating the release of the ParB dimer from the *parS* site and its subsequent sliding on DNA. Consistent with these findings, only CTP-charged *Sc*ParB effectively accumulated on DNA (Fig. 5E).

Surprisingly, we found that the G140S substitution within the CTP-binding motif disrupted the ability of *Sc*ParB^G140S^ to dimerize when studied using BACTH system. It could suggest that G140S substitution might affect the dimerization mediated by CTD. However, taking into account AlphaFold 3 model that did not reveal any disturbances in the interaction between CTDs, we consider this unlikely (Supplementary Fig. S13). On the other hand, the lack of *Sc*ParB^G140S^ dimerization was not detected when analysed *in vitro* using a nonspecific protein cross-linker (glutaraldehyde). We observed that the wild-type and G140S variants form dimer or HMW protein complex as also shown earlier by specific cysteine *Bs*ParB cross-linking [24]. Thus, we speculate that G140S substitution does not impair *Sc*ParB^G140S^ dimer formation itself but may affect the protein conformation altering the spatial orientation of N-terminally fused T18 and T25 subunits. This would prevent the reconstitution of adenylate cyclase activity (but not dimer cross-linking) and may explain the contradiction in the BACTH and protein cross-linking observation.

While CTP binding is crucial for the spread of ParB, the rate of CTP hydrolysis determines the extent to which the ParB protein can migrate beyond the *parS* site. We observed that the CTP–*Sc*ParB complex exhibited no CTP hydrolase activity when not associated with the *parS* site (Fig. 1). This feature appears to be conserved among ParB homologues, serving as an autoinhibitory mechanism that prevents unnecessary CTP utilization by ParB when the protein is not bound to DNA to fulfil its role in segrosome assembly [18, 24]. CTP hydrolysis triggers the opening of the ParB–DNA complex, allowing ParB to be recycled in the cytoplasm (Fig. 5F). Intriguingly, all the ParB homologues are relatively weak CTPases, even in the presence of *parS*-containing DNA. In *S. coelicolor*, ∼1.2 CTP molecules are hydrolysed by *Sc*ParB per minute, which is a hydrolysis rate comparable to that of other ParB homologues [20, 24]. On the other hand, the absence of CTP hydrolysis expands the chromosome region occupied by *Bs*ParB [24]. CTP hydrolysis rate limits the sliding time of ParB on DNA, determining how far from the *parS* site ParB can migrate, thus contributing to the size of the segrosome volume [26, 53, 54]. The complex volumes quantified *in vivo* for the *Corynebacterium glutamicum* producing the wild-type *Cg*ParB protein reached up to 0.05 μm^3^ [29], whereas segrosome volumes detected by us in *S. coelicolor* strain producing the wild-type *Sc*ParB–EGFP were larger, even up to 0.17 μm^3^. We speculate that the increased complex volumes in *S. coelicolor* can be explained by a high number (24 sites) of *parS* sequences [38] in comparison to their number identified in *C. glutamicum* (10 sites) [55]. However, it cannot be excluded that the methanol fixation used by us in sample preparation (but not used in *C. glutamicum* studies) interrupts the quantification of the segrosome complex volume by stabilizing *Sc*ParB interactions or affecting EGFP fluorescence intensity.

The results of our *in vitro* studies on CTP-dependent *Sc*ParB accumulation on DNA are in agreement with the observation that the complex formed *in vivo* by wild-type *Sc*ParB is significantly larger (in terms of volume) and exhibits more intense fluorescence than those formed by all the GERR-substituted *Sc*ParB–EGFP variants. Although our findings confirm the proposed model that DNA and CTP binding are essential for large segrosome formation in *S. coelicolor*, it remains unclear why the *Sc*ParB^R142A^–EGFP variant retained, at least partially, the capacity for partition complex formation. The possibility that the increased dimerization strength observed for *Sc*ParB^R142A^–EGFP partially complements the lack of CTP-dependent accumulation on DNA cannot be excluded. On the other hand, our SIM analyses revealed that the *Sc*ParB^HTH^–EGFP variant also forms complexes with larger volume and intensity than the GERR-substituted variants. Recently, it has been shown that liquid–liquid phase separation is a feature of ParB [29, 30]. *In vitro*,*C. glutamicum Cg*ParB is able to separate into liquid-like droplets, and the phase separation is stimulated by CTP and *parS* binding. The *Cg*ParB^R175A^ variant, which carries a mutation within the Arg patch, was reported to be defective in condensation. In the strain producing *Cg*ParB^R175A^, the foci, although still detectable, were significantly smaller than those in the wild-type strain [29]. It was suggested that phase separation of *Cg*ParB is promoted by CTP binding in a highly crowded environment even in the absence of binding to *parS* [29]. Thus, we speculate that the appearance of ParB^HTH^-EGFP foci in the SIM experiment may be at least partially explained by the phase separation of *Sc*ParB not bound to DNA in comparison to GERR-substituted variants that interact with DNA but not with CTP.

Finally, our studies revealed that substitution within the GERR and HTH motifs affects sporogenic development. Intriguingly, the impact of particular *Sc*ParB variants on chromosome segregation, hyphal growth, and septation was variable and dependent on the introduced modification. The *Sc*ParB^R142A^–EGFP variant, which was defective in CTP binding and hydrolysis, presented very few chromosome segregation defects, which were comparable to those of the wild-type strain. It corroborates the observation that the *S. coelicolor* strain producing the *Sc*ParB^R142A^–EGFP variant still exhibits detectable fluorescent foci when analysed with standard epifluorescence microscopy, but these foci are very rare and randomly positioned compared with those of the wild-type *Sc*ParB–EGFP (Fig. 3A). Although SIM microscopy did not confirm the significant difference of R142A-EGFP foci in comparison to other GERR-substituted *Sc*ParB variants, the glutaraldehyde cross-linking showed the enhanced ability for protein–protein interactions *in vitro*, reinforcing the observation with BACTH system or standard epifluorescence microscopy (Fig. 3 and Supplementary Fig. S11C).

For the strains in which *Sc*ParB–EGFP foci were not detected, the percentage of anucleate spores increased from 7.6% (*Sc*ParB^HTH^–EGFP and *Sc*ParB^G140S^–EGFP variants) to 12.4% (*Sc*Par^G140S;R142A^–EGFP) but was still lower than that quantified for the *parB* deletion strain (16.2%). These observations suggest a more pleiotropic role of the *Streptomyces* ParB homologues in chromosome partitioning. We speculate that *Sc*ParB variants in which segrosome formation was abolished may still retained some of their cellular functions, i.e. interaction with *parS in vitro* (GERR-substituted variants) interaction with ParA, or with SMC, which upon binding to *parS* could partially support chromosome partitioning [35, 41]. The variation in phenotypes of the strains producing different *Sc*ParB variants suggests that the GERR substitutions could have affected different functions of *ScParB*. Interestingly, in *B. subtilis* substitution within *Bs*ParB CTP-binding motif also leads to the observation that both *Bs*ParB^G77S^ and *Bs*ParB^R79A^ variants are abolished in foci formation *in vivo*, however only the G77S but not R79A variant retains its ability to condense DNA *in vitro*. On the other hand, *Bs*ParB^R82A^ variant disrupted in DNA compaction still formed weak foci, suggesting at least partial capacity to segrosome complex formation [8].

As reported earlier, despite its canonical role as a partner for ParA, ParB homologues have been shown to interact with other cellular proteins [56–59, 60] or can contribute to regulation of gene transcription by ParB–*parS* nucleation and local spreading along DNA [61]. However, at the moment other protein partners for *Streptomyces* ParBs (except ParA [41] and SMC [43, 62]) are unknown. Additionally, the role of *Sc*ParB as a regulatory protein involved in sporogenic development has been also reported previously. In *S. venezuelae*, the lack of *Sv*ParB or *Sv*ParA proteins resulted in slower or accelerated tip extension, respectively [35]. Intriguingly, hyphal elongation is not associated with the ability of *Sc*ParB to bind to DNA or form a segrosome since the production of the *Sc*ParB^HTH^–EGFP variant resulted in a similar sporogenic cell length as that observed in the wild-type strain. On the other hand, the GERR substitutions affected the cell length but in a variable manner. While the G140S substitution resulted in shorter sporogenic compartments, similar to *parB* deletion in *S. venezuelae* [35], the G140R;R142A double amino acid substitution led to a significant increase in hyphal length. This could be explained by the impact of the introduced mutations on ParA activity. However, BACTH analysis indicated that the modifications introduced in *Sc*ParB did not affect its interaction with *Sc*ParA.

In summary, our findings show that *S. coelicolor* ParB binds and hydrolyses CTP, which promotes segrosome complex assembly. Since GERR motif substitutions, responsible for CTP binding, lead to hyphae growth, septation, and sporulation defects in *S. coelicolor*, it suggests an involvement of *Sc*ParB in regulatory network controlling *Streptomyces* differentiation and opens a way to study its broader, unexplored role in *S. coelicolor* sporogenic hyphal growth.

## Supplementary Material

gkaf623_Supplemental_Files

## Data Availability

The data underlying this article are available in the article and in its online supplementary material. The fluorescence microscopy data are available in the RODBUK Research Data Repository at https://doi.org/10.34616/L7II3V.

## Notes

Present address: Department of Cell Biology, University of Oklahoma Health Sciences Center, 73104 Oklahoma City, OK, United States
