# Supplementary material for: Significance of the CTP-binding motif for the interactions of S. coelicolor ParB with DNA, chromosome segregation, and sporogenic hyphal growth
Source: Nucleic Acids Res. 2025 Jun 30;53(12):gkaf623. doi: 10.1093/nar/gkaf623 (PMC12207404; doi:10.1093/nar/gkaf623)
Supplement: gkaf623_Supplemental_Files [file gkaf623_supplemental_files.zip › Supplementarly Materials - REVISION.pdf]

SUPPLEMENTARY MATERIALS AND METHODS

Growth rate analysis

To obtain *S. coelicolor* growth curves, we utilised the Bioscreen C instrument (Growth Curves, US). *S. coelicolor* cell cultures were established in sterile honeycomb microplates (Growth Curves, US) containing 300 µl of liquid 79 medium inoculated with 6 × 10^3 colony-forming units (CFUs). Data collection and analysis were conducted according to the general protocol previously described for *S. venezuelae* (36).

Supplementary Table 1. *Escherichia coli* strains used in the study.

Strain name	Relevant genotype and characteristics	Source
DH5α	*F-, Φ80dlacZΔM15, recA1, endA1, gyrA96, thi-E1, hsdR17, (rk-,mk+), supE44, relA1, deoR, Δ(lacZYA-argF)U169*	Laboratory stock
ET12567/pUZ8002	*dam, dcm, hsdS,* CmR, TetR, pUZ8002*: tra,* KanR*, RP4 23;*	(32)
BL21 (DE3) pLysS	F−, *omp*T, *hsd*SB (rB−, mB−), *dcm*, *gal*, λ(DE3), pLysS, CmR	Promega (US)
BW25113/pIJ790	K12 derivative; *araBAD,rhaBAD* λ-Red (*gam bet exo) cat araC rep101*(Ts)	(33)
BTH101	*F-, cya-99, araD139*, *galE15*, *galK16, rpsL1 (*StrR*), hsdR2, mcrA1, mcrB1*	(34)

Supplementary Table 2. *Streptomyces coelicolor* strains used in the study.

Strain	Relevant genotype and characteristics	Source
**M145 (wild-type)**	*S. coelicolor* M145, SCP1-, SCP2-	(35)
**J3303 (*ΔparB*)**	M145 *ΔparB::accIV*	This study
**J3316 (*Sc*ParB^{HTH}-EGFP)**	M145 *parB(IGR(207-209)TYE)-egfp*	(33)
**JS01 (*Sc*ParB^{G140S}-EGFP)**	M145 *parB(G140S)-egfp*	This study
**JS02 (*Sc*ParB^{R142A}-EGFP)**	M145 *parB(R142A)-egfp*	This study
**JS04 (*Sc*ParB$^{G140S;R142A}$-EGFP)**	M145 *parB(G140S;R142A)-egfp*	This study

**JS06 (*Sc*ParB-EGFP)**	M145 *parB-egfp*	This study

Supplementary Table 3. Oligonucleotides used in the study.

Oligonucleotide	Sequence
ScParB_inside_Fw	GTTTCACGTGAAACCGAAG
ScParB_inside_Rv	GAAGAAGCTTCTCGTCCTC
ParB_EcoRI_Rv	GGGAATTCTCAGGACTCGGCGTCCCC
ParB_XbaI_Fw	GTTCTAGACCCAGTGAGTGAGCGACGGAGGGGG
ParB_KpnI_Rv	ATGGTACCATCATATGATTCAGGACTCGGCGTCCCC
EMSA_*parS*_Fw [1)]	CCGAGCTCGAATTCACTAGTGA TTCCG
EMSA_2x_*parS*_Rv	GTCGCGGCAGCCCCTCGC
BLI_*parS*300_Fw [2)]	GGTAGGTTATCCACGTGTTACTC
BLI_*parS*300_Rv [3)]	GAACCAGTGAGGCCTGGTCTTC
*parS*_34pz_Fw	GTGCATCGTGTTTCACGTGAAACGTCGCTCACTG
*parS*_34pz_Rv	CAGTGAGCGACGTTTCACGTGAAACACGATGCAC
mut*parS*_34pz_Fw	GTGCATCGTGTTTCTAGGGAAACGTCGCTCACTG
mut*parS*_34pz_Rv	CAGTGAGCGACGTTTCCCTAGAAACACGATGCAC

[1)] The oligonucleotide was 5’-conjugated with cyanine 3 (Cy3) or cyanine 5 (Cy5) depending on the amplified DNA (see the EMSA experiment description).

[2)] The oligonucleotide was 5’-biotinylated

[3)] The oligonucleotide was 5’-biotinylated only for amplification of DNA biotinylated at both ends (see the BLI experiment description).

Supplementary Table 4. Cosmids and plasmids used in this study

Plasmid	Relevant genotype or characteristics	Source

**H24_*parB* HTH-*egfp***	SuperCos based cosmid containing fragment of *S. coelicolor* chromosome with *parB* gene fused to e*gfp* and modified with *SnaBI* restriction site disrupting nucleotide sequence within the helix-turn-helix HTH motif	(27)
**pGEX-6P-1_*parB***	pGEX-6P-1 derivative encoding the *S. coelicolor parB* gene with in frame 5'-fusion with GST-encoding sequence	(26)
**pGEX-6P-1_*parB*(G140S)**	pGEX-6P-1_*parB* derivative, guanine(418)-to-adenine substitution in the *parB* gene	This study
**pGEX-6P-1_*parB*(R142A)**	pGEX-6P-1_*parB* derivative, cytosine(424)-to-guanine and guanine(425)-to-cytosine substitutions in the *parB* gene	This study
**pGEX-6P-1_*parB*(R143)**	pGEX-6P-1_*parB* derivative, cytosine(427)-to-guanine and guanine(428)-to-cytosine substitutions in the *parB* gene	This study
**pGEX-6P-1_*parB*(G140S;R142A)**	pGEX-6P-1_*parB* derivative, guanine(418)-to-adenine, cytosine(424)-to-guanine and guanine(425)-to-cytosine substitutions in the *parB* gene	This study
**pGEX-6P-1_*parB*(R142A;R143A)**	pGEX-6P-1_*parB* derivative, cytosine(424)-to-guanine and guanine(425)-to-cytosine, cytosine(427)-to-guanine, guanine(428)-to-cytosine substitutions in the *parB* gene	This study
**pGEX-6P-1_*parB*(HTH)**	pGEX-6P-1_*parB* derivative, encoding the *S. coelicolor parB* gene with *SnaBI* restriction site disrupting nucleotide sequence within the helix-turn-helix HTH motif	This study
**pUC19_A7**	pUC19 derivative containing 500 bp fragment of *S. coelicolor parAB* operon upstream region containing two wild-type *parS* sites	(36)
**pUC19_B2**	pUC19 derivative containing 500 bp fragment of *S. coelicolor parAB* operon upstream region containing two scrambled *parS* sites	(36)
**pUT18C**	pUC19 derivative encoding a fragment (T18 domain) of adenylate cyclase(Cya) from *Bordetella pertussis*	(34)
**pKT25**	pSU40 derivative encoding a fragment (T18 domain) of adenylate cyclase (Cya) from *Bordetella pertussis*	(34)
**pUT18C_*parB***	pUT18C derivative encoding the *S. coelicolor* wild-type *parB* gene	This study
**pUT18C_*parB*(140S)**	pUT18C derivative, encoding the *S. coelicolor parB* gene with guanine(418)-to-adenine substitution	This study
**pUT18C_*parB*(142A)**	pUT18C derivative, encoding the *S. coelicolor parB* gene with cytosine(424)-to-guanine and guanine(425)-to-cytosine substitutions	This study

**pUT18C_*parB*(143A)**	pUT18C derivative, encoding the *S. coelicolor* *parB* gene with cytosine(427)-to-guanine and guanine(428)-to-cytosine	This study
**pUT18C_*parB*(G140S;R142A)**	pUT18C derivative, encoding the *S. coelicolor* *parB* gene with guanine(418)-to-adenine, cytosine(424)-to-guanine and guanine(425)-to-cytosine substitutions	This study
**pUT18C_*parB*(R142A;R143A)**	pUT18C derivative, encoding the *S. coelicolor* *parB* gene with cytosine(424)-to-guanine and guanine(425)-to-cytosine, cytosine(427)-to-guanine, guanine(428)-to-cytosine substitutions	This study
**pUT18C_*parB*(HTH)**	pUT18C derivative, encoding the *S. coelicolor* *parB* gene with *SnaBI* restriction site disrupting nucleotide sequence within the helix-turn-helix HTH motif	This study
**pUT18C_parA**	pUT18C derivative encoding the *S. coelicolor* wild-type *parA* gene	(29)
**pKT25_*parB***	pKT25 derivative encoding the *S. coelicolor* wild-type *parB* gene	This study
**pKT25_*parB*(140S)**	pKT25 derivative, encoding the *S. coelicolor* *parB* gene with guanine(418)-to-adenine substitution	This study
**pKT25_*parB*(142A)**	pKT25 derivative, encoding the *S. coelicolor* *parB* gene with cytosine(424)-to-guanine and guanine(425)-to-cytosine substitutions	This study
**pKT25_*parB*(143A)**	pKT25 derivative, encoding the *S. coelicolor* *parB* gene with cytosine(427)-to-guanine and guanine(428)-to-cytosine	This study
**pKT25_*parB*(G140S;R142A)**	pKT25 derivative, encoding the *S. coelicolor* *parB* gene with guanine(418)-to-adenine, cytosine(424)-to-guanine and guanine(425)-to-cytosine substitutions	This study
**pKT25_*parB*(R142A;R143A)**	pKT25 derivative, encoding the *S. coelicolor* *parB* gene with cytosine(424)-to-guanine and guanine(425)-to-cytosine, cytosine(427)-to-guanine, guanine(428)-to-cytosine substitutions	This study
**pKT25_*parB*(HTH)**	pKT25 derivative, encoding the *S. coelicolor* *parB* gene with *SnaBI* restriction site disrupting nucleotide sequence within the helix-turn-helix HTH motif	This study
