# Supplementary material for: Significance of the CTP-binding motif for the interactions of S. coelicolor ParB with DNA, chromosome segregation, and sporogenic hyphal growth
Source: Nucleic Acids Res. 2025 Jun 30;53(12):gkaf623. doi: 10.1093/nar/gkaf623 (PMC12207404; doi:10.1093/nar/gkaf623)
Supplement: gkaf623_Supplemental_Files [file gkaf623_supplemental_files.zip › Supplementary_Figures REVISION2.pdf]

```
Cce      MESVVVGEPGMSEGRRGLGRGLSALLGEVDAAPAQ-----------APG-----------   38
Sco      ----------MSERRRGLGRGLGALIPNAPTEKSVASAALGSAASAAPGAMPLLPNERGV   50
Bsu      -------------MAKGLGKGINALF----------------------------------   13
                        :***:*:.**:

Cce      -----------------------------EQLGGSREAPIEILQRNPDQPRRTFREEDLE   69
Sco      AAAKVATLQHVSRETEELTAPQGVEGLRPPMGAHFAEVPLDAITPNPKQPRKDFDDDALA   110
Bsu      --------NQ-----------------VDLSEETVEEIKIADLRPNPYQPRKHFDDEALA   48
                                             *  :  :  ** ***: * :: *

Cce      DLSNSIREKGVLQPILVRPSPDTAGEYQIVAGERRWRAAQRAGLKTVPIMVRELDDLAVL   129
Sco      ELVTSIREVGLLQPVVVRPT--EPGRYELIMGERRFRACRELELDAIPAIVRATEDEKLL   168
Bsu      ELKESVLQHGILQPLIVRKS--LK-GYDIVAGERRFRAAKLAGLDTVPAIVRELSEALMR   105
         :*  *: : *:***::** :      *::: ****:**.:   *.::* :**  .:  :

Cce      EIGIIENVQRADLNVLEEALSYKVLMEKFERTQENIAQTIGKSRSHVANTMRLLALPDEV   189
Sco      LDALLENLHRAQLNPLEEAFAYDQLLKDFNCTHDQLADRIGRSRPQVSNTLRLLKLSPKV   228
Bsu      EIALLENLQREDLSPLEEAQAYDSLLKHLDLTQEQLAKRLGKSRPHIANHLRLLTLPENI   165
           .::**::* :*. **** :*. *::.:: *::::*. :*:** :::* :*** *  ::

Cce      QSYLVSGELTAGHARAIAAAADPV---ALAKQIIEGGLSVRETEALARKAPNLSA--GKS   244
Sco      QNRVAAGVLSAGHARALLSVDDPEEQDRLAHRIVAEGLSVRSVEEIVTLMGSRPQKPQRA   288
Bsu      QQLIAEGTLSMGHGRTLLGLKNKNKLEPLVQKVIAEQLNVRQLEQLIQQLNQNVPRETKK   225
         *. :. * *: **.*:: .  :      *.::::   *.**. * :     .      :

Cce      KGGRPPRVKDTDTQALESDLSSVLGLDVSIDHRGSTGTLTITYATLEQLDDLCNRLTRGI   304
Sco      KGPRAGSLVSPALSDLATRLSDRFETRVKVDLGQKKGKITVEFASMDDLERILGSLAPGE   348
Bsu      KEPVKD----AVLKERESYLQNYFGTTVNIKRQKKKGKIEIEFFSNEDLDRILELLSERE   281
         *            .   : *.. :   *.:.   ..*.: : : : ::*: :   *:

Cce      --------------------   304
Sco      GPVLQKGLLEGEDEDGDAES   368
Bsu      S-------------------   282
```

Supplementary Figure 1. Alignment of ParB homologues. The amino acid sequences of the ParB homologues were retrieved from the UniProtKB database. The accession numbers for each protein are as follows: *C. crescentus* (B8GW30, *Cce*), *B. subtilis* (P26497, *Bsu*), and *S. coelicolor* (Q9RFN2, *Sco*). The ParA-binding motif is highlighted in red, the CTP-binding motif in blue, and the helix-turn-helix (HTH) motif in green. The amino acids substituted in the *Sc*ParB variants described in this study are indicated by a red line.

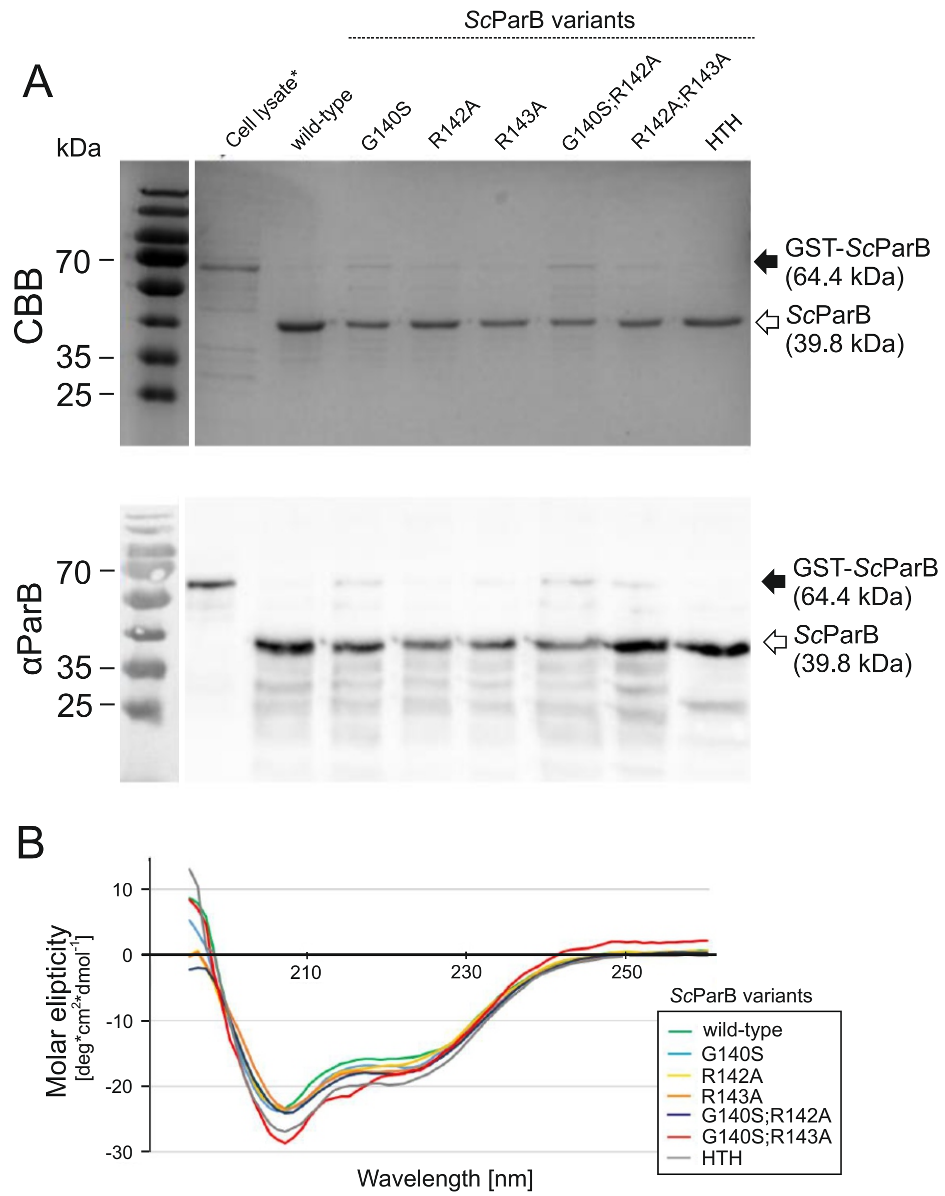

Supplementary Figure 2. Purification of *Sc*ParB variants. (A) Analysis of purified *Sc*ParB variants (wild-type, G140S, R142A, R143A, G140S;R142A, R142A;R143A, and HTH) using SDS–PAGE, followed by staining with Coomassie Brilliant Blue (CBB) or Western blotting with anti-ParB serum (αParB). An example of a cell lysate obtained from IPTG-induced *E. coli* BL21 (DE) pLysS cells producing wild-type GST-*Sc*ParB (64.4 kDa, black arrow) is shown (cell lysate). The purified recombinant *Sc*ParB variants after cleavage with the PreScission protease (39.8 kDa, white arrow) are indicated according to the amino acid substitutions. The 25, 35 and 70 kDa bands of the protein molecular weight ladder are marked on the left. (**B**) Normalised circular dichroism (CD) spectra of *Sc*ParB variants (wild-type, G140S, R142A, R143A, G140S;R142A, R142A;R143A, and HTH). The CD spectra were recorded in triplicate, and the average molar ellipticity [deg * cm^2 *$dmol^{-1}$] was plotted against the wavelength [nm].

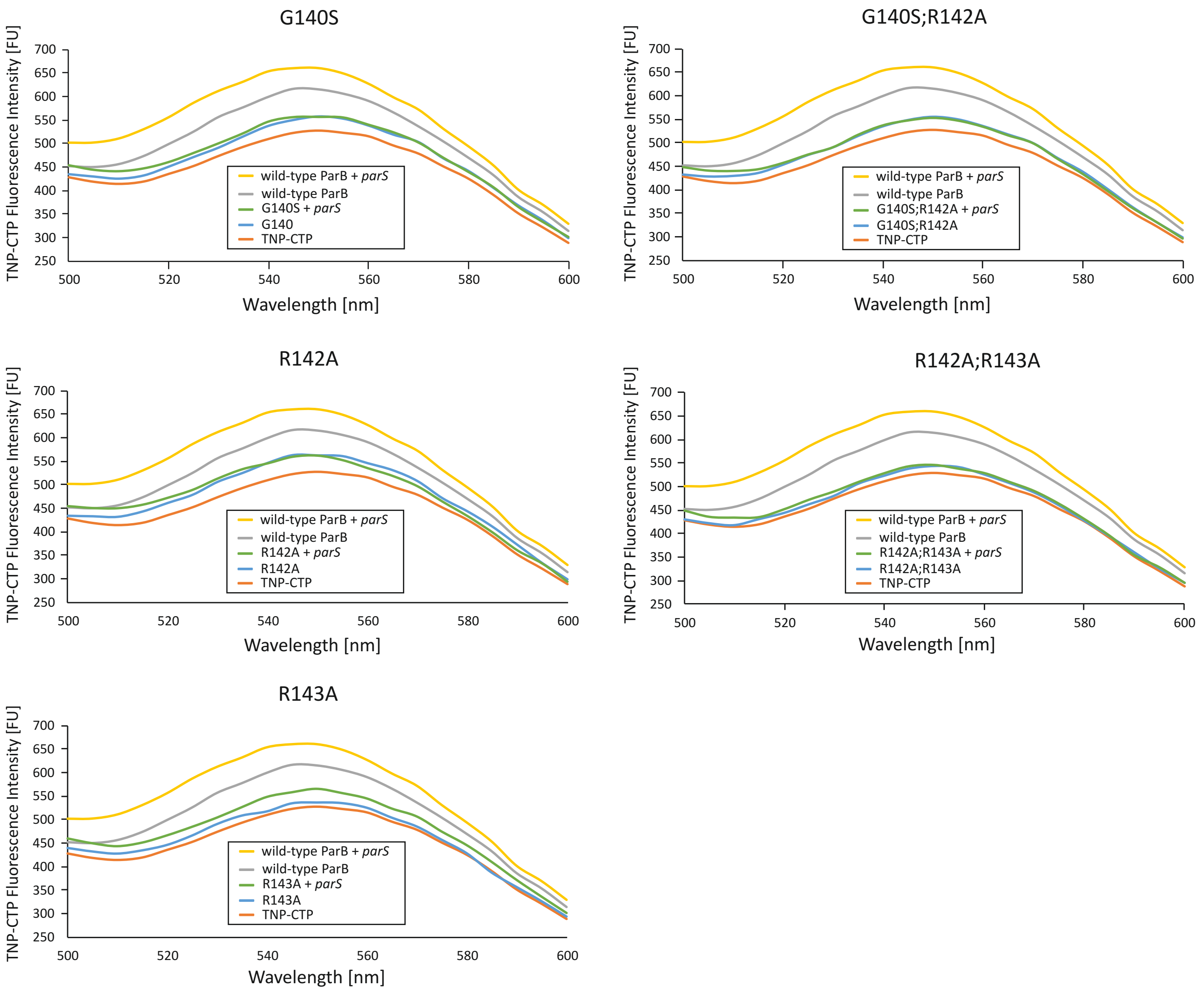

Supplementary Figure 3. TNP-CTP binding by GERR-substituted *Sc*ParB variants. The fluorescence intensity ([FU] spectra of 5 µM TNP-CTP measured after binding to specific *Sc*ParB variants at a concentration of 1 µM (G140S, R142A, R143A, G140S;R142A, R142A;R143A). Each analysis was conducted in the presence (green) or absence (blue) of a 34-bp *parS*-containing DNA fragment (2 µM) and compared with spectra recorded for the wild-type *Sc*ParB (yellow and grey, respectively, in the presence or absence of DNA) or a 5 µM TNP-CTP sample with no protein added (orange).

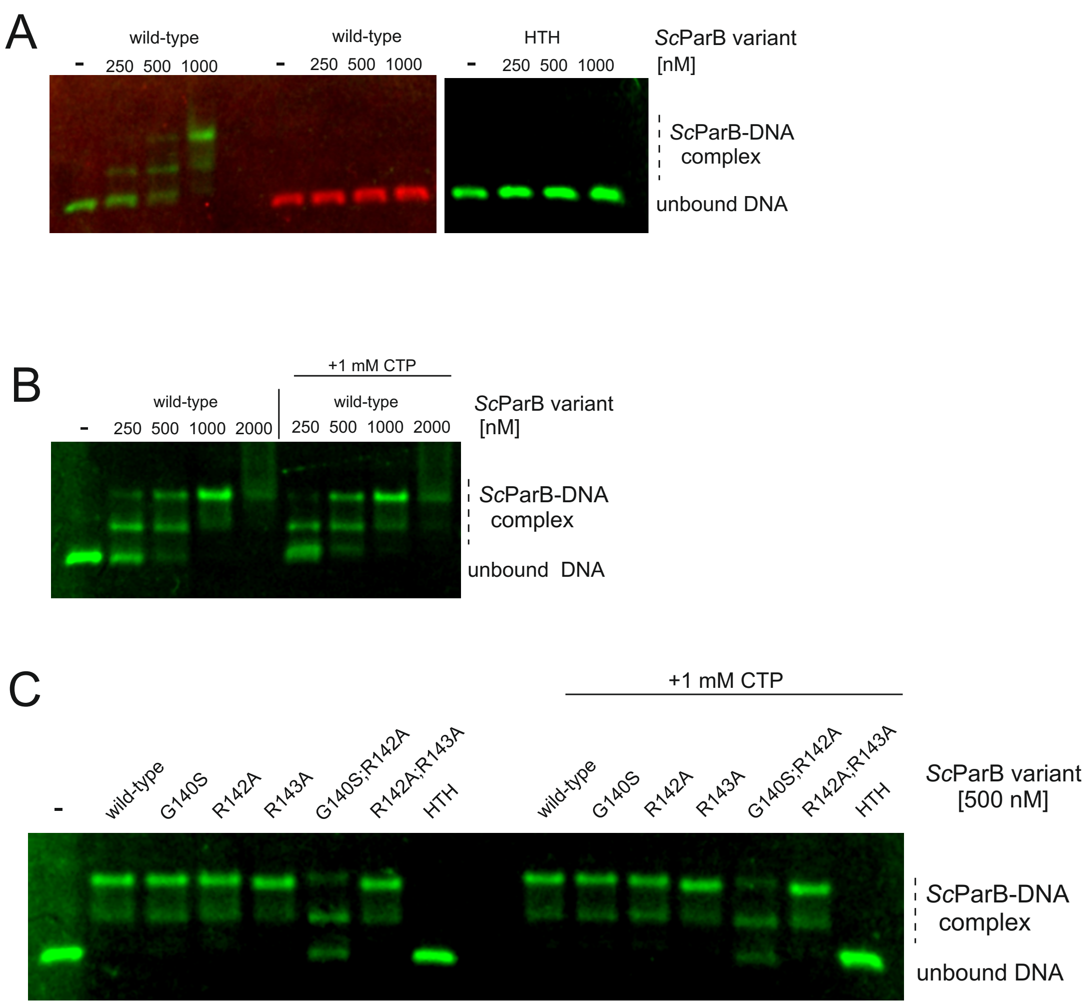

Supplementary Figure 4. Impact of CTP on *Sc*ParB-parS binding *in vitro* (**A**) *Left*: Analysis of recombinant wild-type *Sc*ParB (250–1000 nM) binding to a 500-bp Cy5-labelled (green) linear DNA fragment (10 µM) containing two *parS* sites or a Cy3-labelled (red) DNA fragment with two scrambled *parS* sites. *Right*: Analysis of the binding of the non-DNA-interacting *Sc*ParB variant (HTH) (250–1000 nM) to the 500-bp Cy5-labelled linear (green) DNA fragment (10 µM) containing two *parS* sites. (**B**) Analysis of recombinant wild-type *Sc*ParB (250–1000 nM) binding to the 500-bp Cy5-labelled (green) linear DNA fragment (10 µM) containing two *parS* sites in the presence (right) or absence (left) of 1 mM CTP. (**C**) Analysis of recombinant *Sc*ParB variants (wild-type, G140S, R142A, R143A, G140S;R142A, R142A;R143A, and HTH) at 500 nM in the 500-bp Cy5-labelled (green) linear DNA fragment (10 µM) containing two *parS* sites in the presence (right) or absence (left) of 1 mM CTP. The unbound DNA and *Sc*ParB-parS complexes are indicated on the right.

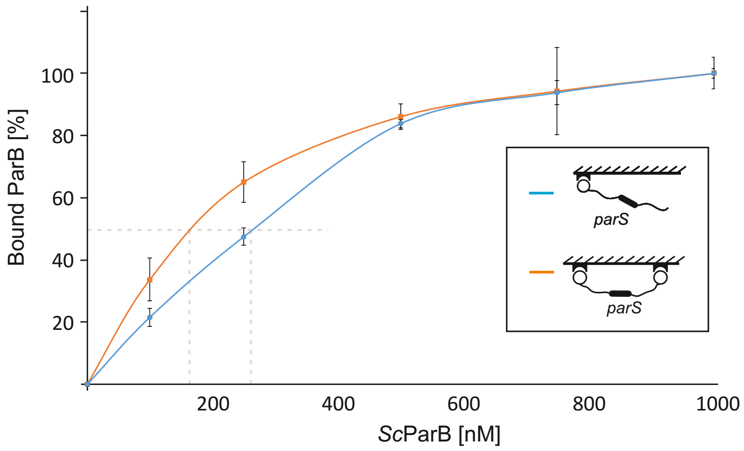

Supplementary Figure 5. Quantification of the dissociation constant (K_d) of the *Sc*ParB-*parS* complex. The Kd values were calculated on the basis of BLI measurements conducted for one- (blue) or two-end (orange) immobilised *parS*-containing DNA at the steady state of protein complex formation. The percentage of bound DNA was plotted against the *Sc*ParB concentration.

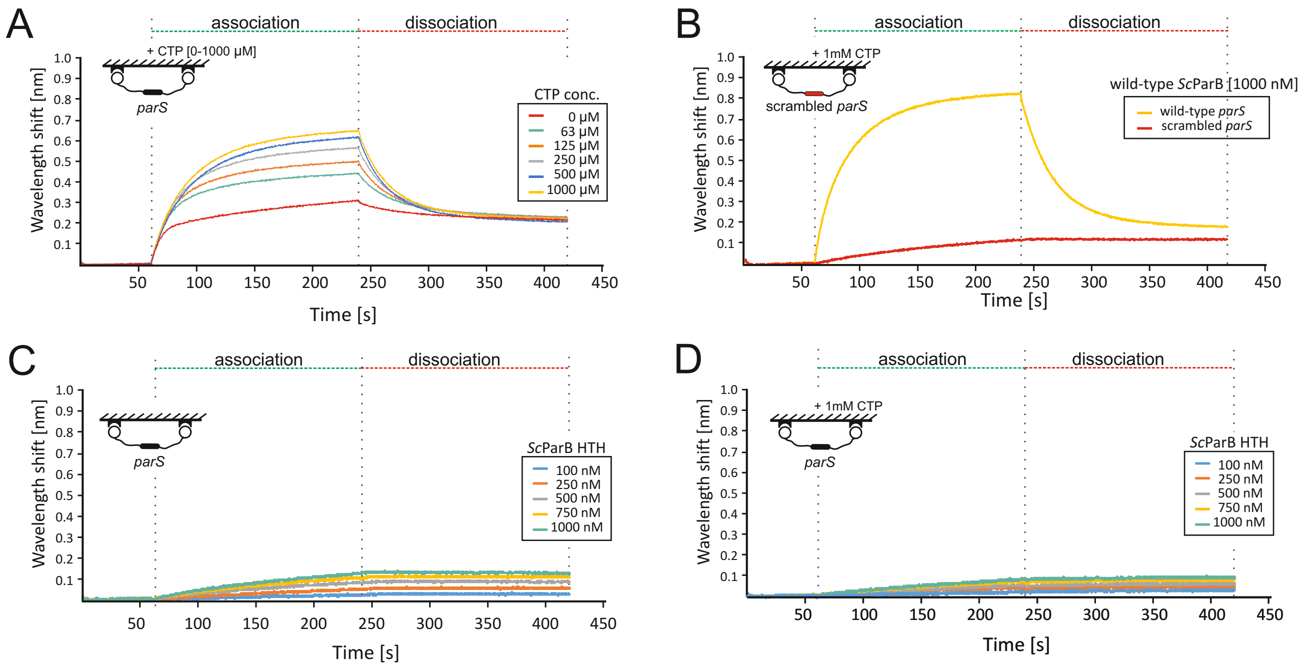

Supplementary Figure 6. Analysis of *Sc*ParB interactions with a two-end biotin-immobilised 300-bp DNA fragment using BLI. (A) Binding of wild-type *Sc*ParB (1000 nM) to a DNA fragment containing a wild-type *parS* site (yellow) or a scrambled *parS* site in the presence of 1 mM CTP. (**B**) Binding of wild-type *Sc*ParB (500 nM) to a DNA fragment containing a *parS* site conducted in the presence of a broad range of CTP concentrations (63–1000 µM). (**C**) Binding of the non-DNA-interacting *Sc*ParB^{HTH} variant to a DNA fragment containing a wild-type *parS* site over a broad range of protein concentrations (0–1000 nM). (**D**) Binding of the non-DNA-interacting *Sc*ParB^{HTH} variant to a DNA fragment containing a wild-type *parS* at a broad range of protein concentrations (0–1000 nM) in the presence of 1 mM CTP. The association and dissociation steps are indicated by green and red dotted lines, respectively.

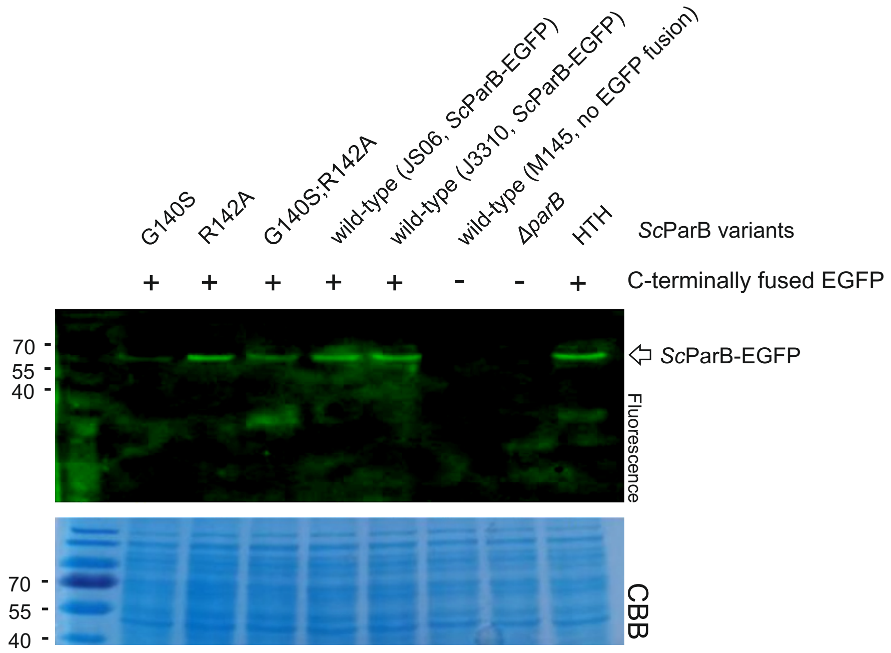

Supplementary Figure 7. Analysis of *Sc*ParB-EGFP variant production in *S. coelicolor*. The fluorescence signal detected in cell lysates obtained from *S. coelicolor* strains producing ScParB-EGFP variants (wild-type, G140S, R142A, G140S;R142A, and HTH). Cell lysates obtained from the wild-type (without EGFP fusion) and *parB* deletion (Δ*parB*) strains were used as controls. The loading control, represented by CBB-stained acrylamide gel (CBB), is displayed below. The 40, 55 and 70 kDa bands of the protein molecular weight ladder are indicated on the left.

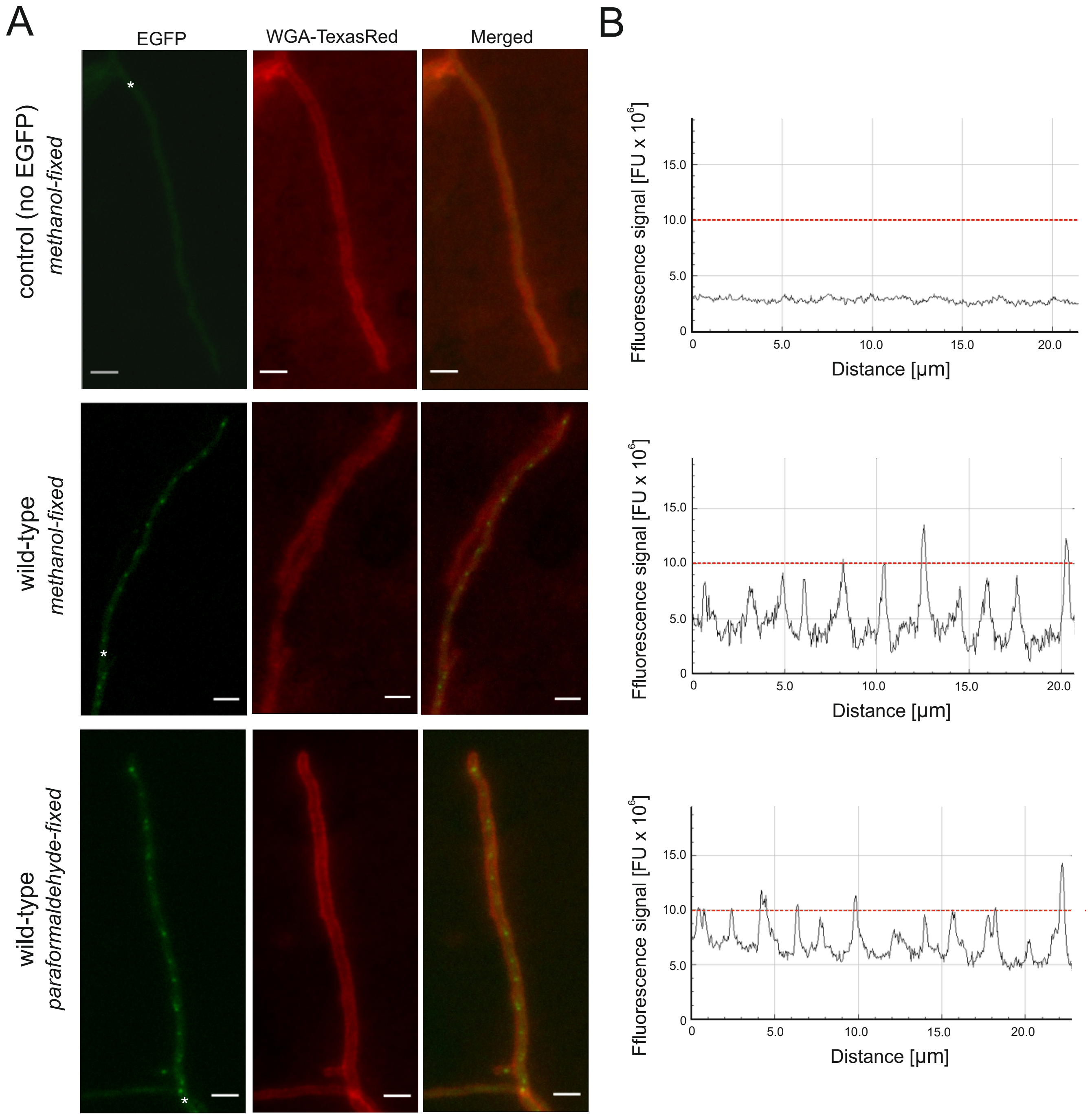

Supplementary Figure 8. Analysis of EGFP fluorescence intensity in *S. coelicolor*. (A) The representative images of *S. coelicolor* sporogenic hyphae producing wild-type *Sc*ParB-EGFP fixed with absolute methanol or 2.8% paraformaldehyde. The peptidoglycan was visualised using wheat germ agglutinin conjugated to Texas Red (WGA-TexasRed). The wild-type *S. coelicolor* strain, which does not produce EGFP-fused *Sc*ParB, served as a control. Scale bar: 2 μm. (**B**) The comparison of EGFP fluorescence signal (FU) along methanol- or paraformaldehyde-fixed sporogenic hyphae quantified using ImageJ software. The starting point of each measurment was marked with a asterisk on the microsope images.

A

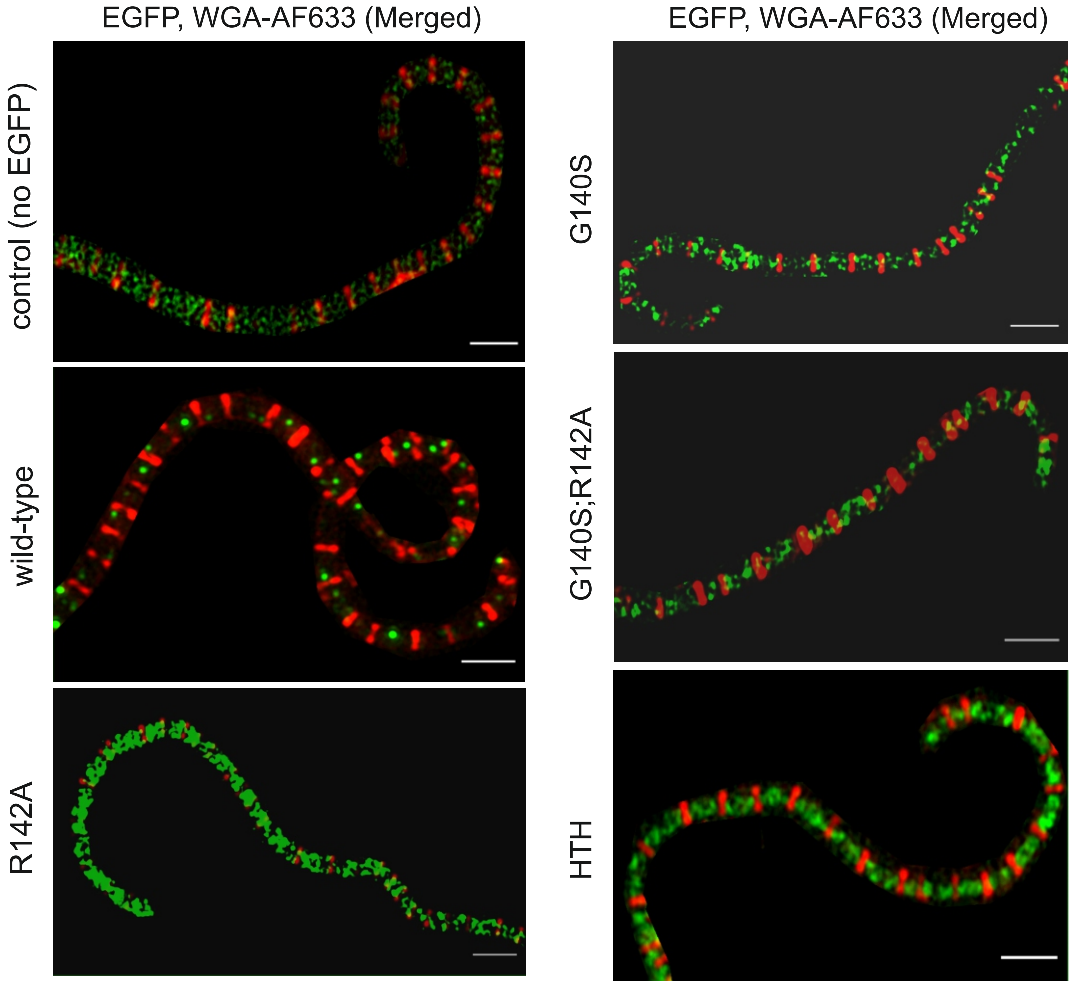

B

Strain	Number of sporogenic cells	Number of detected *Sc*ParB-EGFP foci	Average volume of *Sc*ParB-EGFP complex [μm^3]
Control (no EGFP)	14	11378	0,012
wild-type	19	607	0,062
R142A	19	1452	0,042
G140S	18	2980	0,029
G140S;R14A	19	1525	0,041
HTH	14	1017	0,054

Supplementary Figure 9. Detection of *Sc*ParB-EGFP foci in *S. coelicolor*. (A) The representative processed and overlaid Z-stack images of *S. coelicolor* sporogenic hyphae producing *Sc*ParB-EGFP variants (wild-type, G140S, R142A, G140S;R142A, and HTH) obtained using SIM microscopy. The wild-type *S. coelicolor* strain, which does not produce EGFP-fused *Sc*ParB, served as a control. The peptidoglycan was visualised using wheat germ agglutinin conjugated to Alexa Fluor-633 (WGA-AF633) and merged with *Sc*ParB-EGFP. Scale bar: 2 µm (**B**) The summary of SIM experiment showing the number of analysed sporogenic cells, the number of detected *Sc*ParB-EGFP foci and the average foci volume.

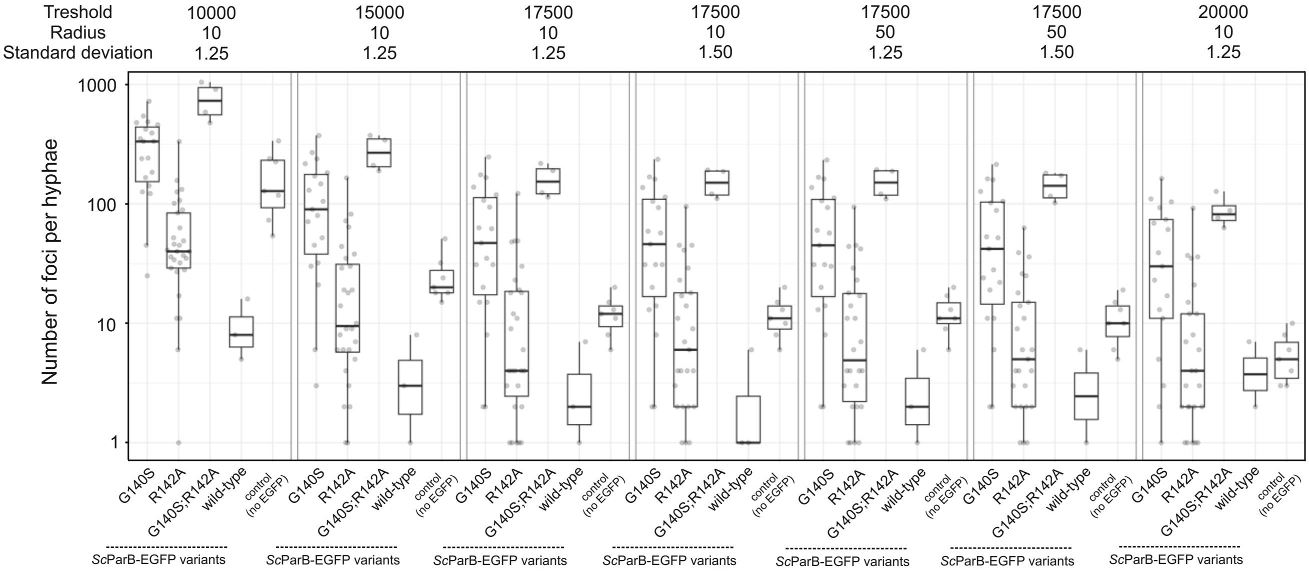

Supplementary Figure 10. Optimization of *Sc*PArB-EGFP foci detection identified in SIM microscopy images. The representative processed images of *S. coelicolor* sporogenic hyphae producing *Sc*ParB-EGFP variants (wild-type, G140S, R142A, G140S;R142A) obtained using SIM microscopy were analysed using Fiji 3D Image Suite package and 3D ‚Local Maxima' function. Each boxblot represents the number of identified EGFP foci per a single hyphae depending on the set up paramteres (treshold, radius, and standard deviation). Each boxplot shows the median with the first and third quartiles, whereas the lower and upper 'whiskers' extend to values no further than 1.5 times the interquartile range.

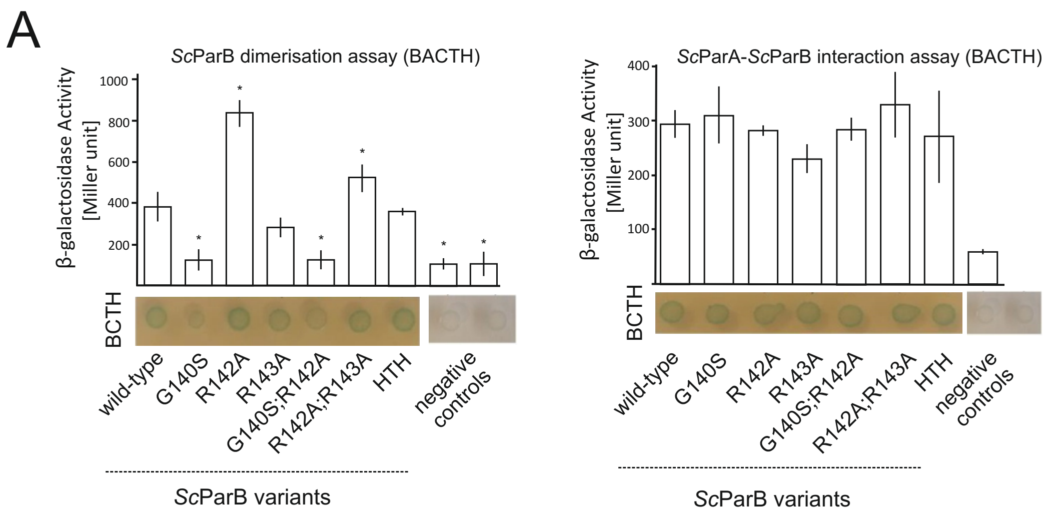

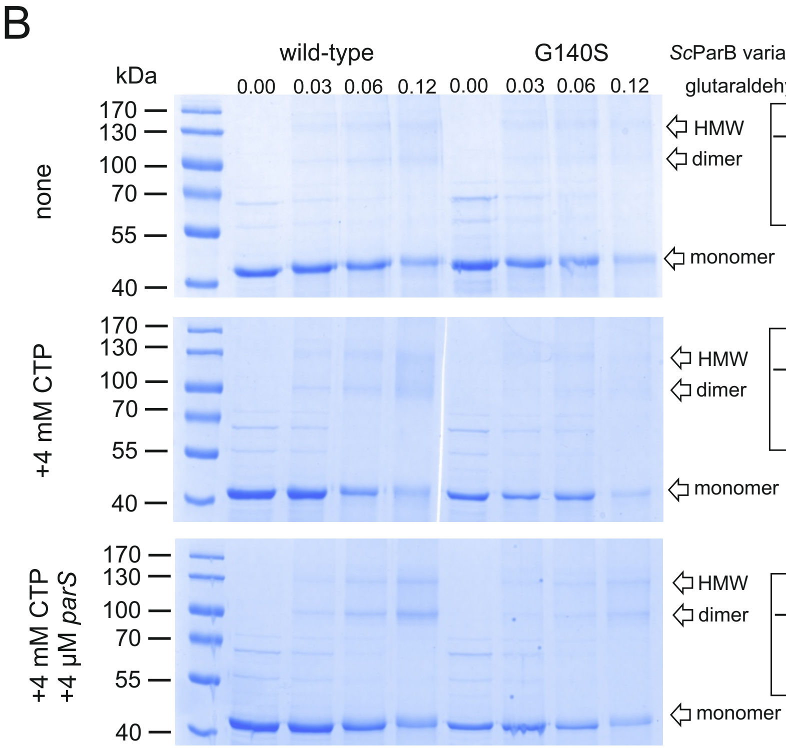

none

*Sc*ParB variant	Total signal [U]	dimer [%]	HMW [%]
WT	221450	3.1	3.2
G140S	202331	1.1	1.6

+0.12 %glutaraldehyde

+ 4 mM CTP

*Sc*ParB variant	Total signal [U]	dimer [%]	HMW [%]
WT	221105	6.4	4.9
G140S	202331	1.6	1.2

+0.12 %glutaraldehyde

+4 mM CTP; +4 µM *parS*

*Sc*ParB variant	Total signal [U]	dimer [%]	HMW [%]
WT	200053	5.3	2.7
G140S	211068	2.1	1.9

+0.12 %glutaraldehyde

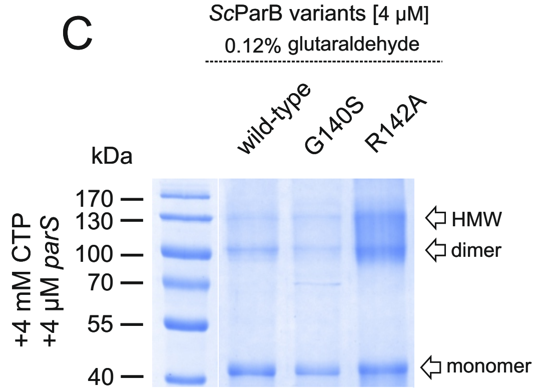

+4 mM CTP; +4 µM *parS*

*Sc*ParB variant	Total signal [U]	dimer [%]	HMW [%]
WT	221471	5.1	2.8
G140S	192003	3.3	2.7
R142A	194600	14.7	12.1

+0.12 %glutaraldehyde

Supplementary Figure 11. Analysis of *Sc*ParB protein interactions. (**A**) Dimerisation capacity (left) or *Sc*ParA interaction (right) of *Sc*ParB variants (wild-type, G140S, R142A, R143A, G140S;R142A, R142A;R143A, and HTH) fused with T25 or T18 adenylate cyclase subunits. For the *Sc*ParA-*Sc*PArB interaction assay, *Sc*ParA was fused with the T18 subunit, whereas *Sc*ParB was fused with the T25 subunit. Protein interactions were quantified using a bacterial two-hybrid (BACTH) system or β-galactosidase activity assay with results expressed in Miller units. The negative controls included *E. coli* BTH101 cells cotransformed with the pKT25 plasmid and wild-type *parA*- or *parB*-expressing plasmids (pUT18C-*parA* or pUT18C-*parB,* respectively) or the pKT25 and pUT18 plasmids. (**B**) The analysis of protein-protein interactions using 0.03 - 0.12 % glutaraldehyde. (**C**) The analysis of protein-protein interactions using 0.12 % glutaraldehyde. The reactions were perfromed for 4 µM *Sc*ParB variants (wild-type, G140S, or R142A) in the presence of CTP (4 mM) and/or 4 µM 34-bp dsDNA fragments containing *parS* sequence (4 µM). The protein molecular weights (kDa) are indicated on the left. The position of *Sc*ParB monomer, dimer and high molecular weight complex (HMW) visualised in CBB-stained acrylamide gel is marked with arrows. The percentage contribution of the observed signal for the dimer and HMW, as well as the total signal readout measured for the sample cross-linked with 0.12 % glutaraldehyde is presented on the right side.

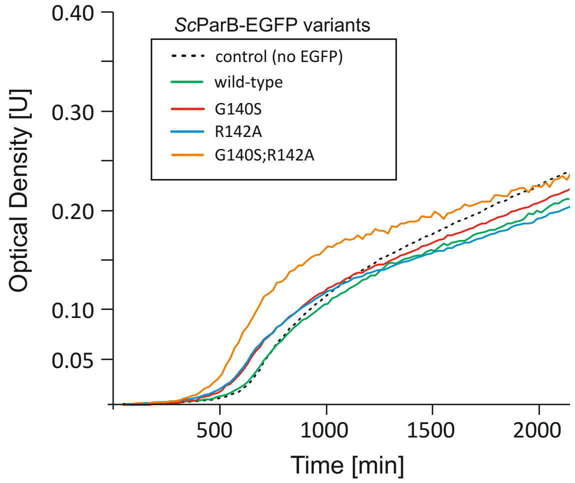

Supplementary Figure 12. Growth of *S. coelicolor* strains producing *Sc*ParB-EGFP variants. The growth was analysed in liquid 79 medium: wild-type [green], G140S [red], R142A [blue], G140S;R142A [orange]. The wild-type *S. coelicolor* strain, which does not produce EGFP-fused *Sc*ParB, served as a control (black dotted line).

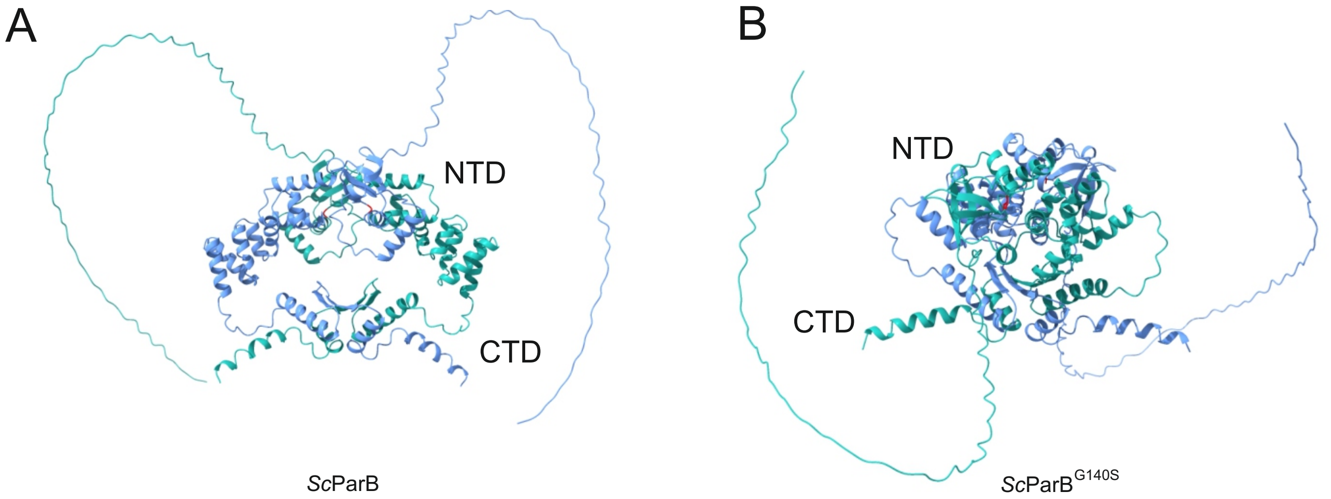

Supplementary Figure 13. AlphaFold 3 prediction of *Sc*ParB homodimer structure. (A) wild-type *Sc*ParB and (B) ScParBG140S variants. Particular *Sc*ParB momomers (green and violet) as well as the position of the NTD and CTD domains are marked. The glycine or serine amino acid residues in position 140 are marked in pink.
